# Monocyte/macrophage-derived interleukin-15 mediates the pro-inflammatory phenotype of CD226^+^ B cells in type 1 diabetes

**DOI:** 10.1016/j.ebiom.2025.105946

**Published:** 2025-09-15

**Authors:** Jingyue Li, Xin Liang, Mingjiu Zhao, Wenjun Luo, Juan Huang, Yang Xiao, Jiaqi Huang, Bin Zhao, Zhiguang Zhou

**Affiliations:** a National Clinical Research Center for Metabolic Diseases, Key Laboratory of Diabetes Immunology, Ministry of Education, and Department of Metabolism and Endocrinology, The Second Xiangya Hospital of Central South University, Changsha, Hunan, China; b CSU-Sinocare Research Center for Nutrition and Metabolic Health, Xiangya School of Public Health, Central South University, Changsha, Hunan, China; c Furong Laboratory, Changsha, Hunan, China; d Section of Endocrinology, Department of Internal Medicine, School of Medicine, Yale University, New Haven, CT, USA

**Keywords:** Type 1 diabetes, Autoimmune diabetes, Combination therapy, B cells, CD226

## Abstract

**Background:**

Type 1 diabetes (T1D) is characterised by the autoimmune-mediated destruction of pancreatic β-cells. Although traditionally viewed as a disease dominated by T cells, recent studies have emphasised the crucial role of B cells in the development of T1D. Genome-wide association studies (GWAS) have revealed that CD226 is related to susceptibility to several autoimmune diseases, including T1D. Our recent work identified a pathogenic role of CD226^+^ CD8^+^ T cells in T1D. However, the involvement of CD226^+^ B cells in T1D development remains unclear.

**Methods:**

The expression and functional characteristics of CD226^+^ B cells in T1D patients and non-obese diabetic (NOD) mice were detected by flow cytometry. RNA sequencing and molecular biology experiments were performed to reveal regulatory mechanisms. In addition, in vivo interventions were conducted to explore potential preventive and therapeutic targets for T1D.

**Findings:**

The percentage of CD226^+^ B cells is increased and positively correlated with disease severity in T1D. CD226^+^ B cells from T1D patients and NOD mice exhibit increased capability for activation, proliferation, and production of pro-inflammatory cytokines along with heightened glycolytic metabolism. Mechanistic studies have revealed that interleukin-15 (IL-15) secreted by monocytes or macrophages promotes the inflammatory response of CD226^+^ B cells. Importantly, the use of an anti-CD132 monoclonal antibody (anti-CD132) or an anti-IL-15 monoclonal antibody (anti-IL-15), which blocks IL-15 signalling, effectively prevented the disease onset of T1D. Furthermore, combination therapy with anti-CD3 monoclonal antibody (anti-CD3) and anti-CD132 synergistically reversed hyperglycemia in cyclophosphamide-accelerated NOD mice.

**Interpretation:**

Our study demonstrates a novel role of the monocyte/macrophage-IL-15-CD226^+^ B cell axis in T1D immunopathogenesis and provides potential targets for T1D immunotherapy.

**Funding:**

This work was supported by the Noncommunicable Chronic Diseases-10.13039/501100018537National Science and Technology Major Project (2023ZD0507300, 2023ZD0507303, 2023ZD0508200, and 2023ZD0508201), the 10.13039/501100001809Natural Science Foundation of China (82570973, 82170795, 82470814, 82100949, and 82470931), the Scientific Research Program of FuRong Laboratory (2024PT5105) and the 10.13039/501100002822Central South University Research Programme of Advanced Interdisciplinary Studies (2023QYJC008).

**Research in context**

**Evidence before this study**

In addition to T cells, an increasing body of evidence suggests that B cells also play an indispensable role in the pathogenesis of type 1 diabetes (T1D). Genome-wide association studies (GWAS) have confirmed that polymorphisms in the CD226 gene are associated with susceptibility to a range of autoimmune diseases, including T1D. Our previous studies have demonstrated the pathogenic role of CD226^+^ CD8^+^ T cells in T1D. Research from other laboratories has also highlighted the pathogenic role of CD226^+^ B cells in systemic lupus erythematosus (SLE). However, the role of CD226^+^ B cells in T1D, their regulatory mechanisms, and their interactions with other immune cells remain to be elucidated.

**Added value of this study**

We first identified an elevated expression of CD226 on B cells in patients with T1D, which positively correlated with disease severity. Mechanistically, we reported that CD226^+^ B cells, under the driver of nuclear factor-kappa B (NF-κB) signalling, exhibit increased activation, proliferation, and production of pro-inflammatory cytokines. In addition, monocyte/macrophage-derived interleukin-15 (IL-15) promotes the generation and pro-inflammatory functions of CD226^+^ B cells. Using patient data, mouse models, and comprehensive in vitro and in vivo experiments, we propose a novel monocyte/macrophage-IL-15-CD226^+^ B cell axis in the pathogenesis of T1D, providing a theoretical foundation for T1D immunotherapies.

**Implications of all the available evidence**

After elucidating the pathogenic role of the monocyte/macrophage-IL-15-CD226^+^ B cell axis in T1D, we further explore effective intervention targets for T1D treatment. We found that anti-CD132 monoclonal antibody (anti-CD132), which blocks IL-15 signalling, not only prevented the onset, but also synergistically reversed hyperglycemia with anti-CD3 monoclonal antibody (anti-CD3) in cyclophosphamide-accelerated NOD mice, which significantly enhances the clinical value of this study.

## Introduction

Type 1 diabetes (T1D) is a chronic metabolic disorder due to autoimmune-mediated breakdown of insulin-producing pancreatic β-cells.^1^ The aetiology of T1D includes a multifaceted interaction of genetic predispositions, environmental elements, and compromised immune responses, which collectively lead to a breakdown in self-tolerance.^2^^,^^3^ While existing immunotherapies especially Teplizumab therapy in T1D have shown promise, they have limitations that are far from meeting the current clinical need for sustaining persistent normoglycemia and reducing insulin dependence.^4^^,^^5^ Thus, in-depth examination of the immunopathogenesis of T1D is essential for immune intervention and other therapeutic strategies to achieve clinical remission of this disorder.

Although T1D is generally regarded as a T-cell-mediated disease,^6^ growing evidence indicates that B cells also play a critical role. B cells can present antigens, secrete cytokines, produce autoantibodies, express co-stimulatory molecules, and contribute to the initiation and proliferation of self-reactive T cells, facilitating the T1D pathogenesis.^7^, ^8^, ^9^, ^10^ Notably, B-cell depletion therapy has shown potential in slowing down the decline of C-peptide levels in individuals with newly diagnosed T1D; however, the protective effect disappears after one year.^11^ This may be due to the incomplete clearance of some mature B cells with low CD20 expression and the deletion of regulatory B cells with protective function. Previous studies, along with work from our laboratory, identified an imbalance in peripheral B cell subsets in T1D, including an increase in pathogenic B cell subsets.^12^^,^^13^ Additionally, we observed a metabolic shift in pro-inflammatory B cells towards increased glucose uptake in T1D.^14^ Therefore, targeting the inflammatory B cell subset may offer novel strategies to restore immune tolerance and develop immunoregulatory therapies for T1D.

Immune checkpoints are pivotal in B cell immune responses regulation. CD226 is a co-stimulatory immune checkpoint, primarily expressing on effector and memory T cells, natural killer (NK) cells, and B cells.^15^^,^^16^ Previous studies have indicated that a single nucleotide polymorphism (SNP) in CD226 (rs763361) is related to various autoimmune disorders, such as T1D, rheumatoid arthritis (RA), celiac disease, and psoriasis.^17^ Earlier researches have indicated that the frequency of CD226^+^ B cells is increased and positively correlated with the severity of systemic lupus erythematosus (SLE).^18^ In addition, our recent work has also identified a pathogenic role of CD226^+^ CD8^+^ T cells in T1D.^19^ Nonetheless, the characteristics of CD226^+^ B cells in T1D, their regulatory mechanisms, and interactions with other immune cells remain to be elucidated.

In this study, we identified elevated CD226 expression on B cells in T1D patients, which was positively related to disease severity. To elucidate the role of CD226^+^ B cells, we conducted functional, metabolic, and transcriptional analyses of CD226^+^ B cells from patient samples and disease animal models. These cells demonstrated increased activation, proliferation and pro-inflammatory cytokine production, driven by nuclear factor kappa-light-chain-enhancer of activated B cells (NF-κB) signalling. Furthermore, our data revealed that interleukin-15 (IL-15), secreted by monocytes or macrophages, promotes the generation and pro-inflammatory function of CD226^+^ B cells. Importantly, an anti-CD132 monoclonal antibody (anti-CD132) or an anti-IL-15 monoclonal antibody (anti- IL-15) prevented the onset of T1D and improved its condition as prophylactic administration in nondiabetic non-obese diabetic (NOD) mice. In addition, anti-CD132 increased the disease remission rate and reduced blood glucose levels and insulitis severity when combined with anti-CD3 monoclonal antibody (anti-CD3) in diabetic NOD mice. In conclusion, our study offers a novel monocyte/macrophage-IL-15-CD226^+^ B cell axis in T1D pathogenesis, which links innate and adaptive immunity, and offers a rationale for the development of new immunotherapies for T1D.

## Methods

### Study participants and ethics statement

Forty T1D patients, forty healthy controls (HC), twenty latent autoimmune diabetes in adults (LADA) patients and twenty type 2 diabetes (T2D) patients in the Second Xiangya Hospital of Central South University entered into our study (Clinical Trials.gov ID: NCT03610984) from February 2022 to July 2023 (Table 1). The inclusion criteria for T1D, LADA, T2D, and HC referred to our previously published article.^14^ The research received approval from the ethical committee of the Second Xiangya Hospital, Central South University. Every participant agreed to take part in this research and offered informed approval.

**Table 1:** Physical and clinical characteristics of the study participants.

	HC (n = 40)	T2D (n = 20)	LADA (n = 20)	T1D (n = 40)
Male (%)	52.50 (21/40)	90.00 (18/20)^b^^,^^i^	70.00 (14/20)	45.00 (18/40)
Age (years)	12.00 (9.00,14.00)	41.80 ± 17.29^d^^,^^k^	43.20 ± 12.98^d^^,^^k^	11.00 (6.50,15.00)
Height (cm)	NA	167.73 ± 7.89^k^	165.13 ± 7.99^j^	151.75 (115.00,164.75)
Weight (kg)	NA	65.98 ± 15.27^k^	61.91 ± 10.65^k^	35.90 (21.55,55.10)
BMI (kg/m^2^)	NA	22.38 (20.59,25.06)^k^	22.65 ± 3.28^k^	18.01 ± 3.34
Duration (months)	NA	48.00 (8.00,120.00)^j^	23.50 (19.25,80.50)	17.50 (4.00,30.88)
FBG (mmol/L)	4.84 ± 0.48	7.51 (6.42,8.92)^d^	7.10 ± 2.92^b^	7.55 ± 2.50^d^
2hBG (mmol/L)	NA	8.37 (7.19,13.73)^h^	14.78 ± 6.43	15.71 ± 5.57
FCP (pmol/L)	NA	545.07 ± 316.88^k^	198.86 ± 136.16^g^	93.10 (57.90,144.03)
2hCP (pmol/L)	NA	861.65 (438.18,3355.28)^k^	353.70 (212.68,1000.25)^e^	250.20 (90.80,472.90)
HbA1c (%)	NA	6.99 (6.36,9.12)	6.92 (6.09,7.74)	7.38 (6.85,8.64)
GA (%)	NA	15.61 (13.30,18.55)	15.95 (15.43,19.22)	18.07 (17.09,20.73)
TC (mmol/L)	3.89 (3.64,4.29)	4.27 ± 1.29	4.29 (3.68,4.60)	4.58 ± 0.97^a^
TG (mmol/L)	1.03 ± 0.54	1.98 (1.05,2.47)^c^^,^^k^	0.86 ± 0.38^f^	0.60 (0.48,0.83)
LDL-C (mmol/L)	1.47 ± 0.35	2.51 ± 0.88^d^	2.41 ± 0.61^d^	2.61 ± 0.69^d^
HDL-C (mmol/L)	2.28 ± 0.53	1.08 ± 0.24^d^^,^^k^	1.43 ± 0.45^d^	1.73 ± 0.41^d^
GADA positive (%)	NA	0.00 (0/20)	90.00 (18/20)	65.00 (26/40)
IA-2A positive (%)	NA	0.00 (0/20)	35.00 (7/20)	80.00 (32/40)
ZnT8A positive (%)	NA	0.00 (0/20)	20.00 (4/20)	52.50 (21/40)
Number of positive autoantibodies (%)				
1	NA	0.00 (0/20)	65.00 (13/20)	32.50 (13/40)
2	NA	0.00 (0/20)	20.00 (4/20)	37.50 (15/40)
3	NA	0.00 (0/20)	15.00 (3/20)	30.00 (12/40)

HC and T2D patients were recruited and matched for sex and age to T1D patients and LADA patients, respectively. The data are expressed as the mean ± standard deviation for normally distributed data and the median (25th, 75th percentiles) for data deviated from normal distribution. One-way ANOVA followed by adjustments was used for multiple comparisons. Abbreviations: HC, healthy control; T1D, type 1 diabetes; LADA, latent autoimmune diabetes in adults; T2D, type 2 diabetes; BMI, body mass index; FBG, fasting blood glucose; 2hBG, 2-hour postprandial blood glucose; FCP, fasting C-peptide; 2hCP, 2-hour postprandial C-peptide; HbA1c, glycated haemoglobin; GA, glycated albumin; TC, total cholesterol; TG, triglyceride; LDL-C, low density lipoprotein-cholesterol; HDL-C, high density lipoprotein-cholesterol; GADA, glutamic acid decarboxylase antibody; IA-2A, protein tyrosine phosphatase antibody; ZnT8A, zinc transporter 8 antibody; NA, not appropriate.

a P < 0.05 compared with HC.

b P < 0.01 compared with HC.

c P < 0.001 compared with HC.

d P < 0.0001 compared with HC.

e P < 0.01 compared with T2D patients.

f P < 0.001 compared with T2D patients.

g P < 0.0001 compared with T2D patients.

h P < 0.05 compared with T1D patients.

i P < 0.01 compared with T1D patients.

j P < 0.001 compared with T1D patients.

k P < 0.0001 compared with T1D patients.

### Extraction of peripheral blood mononuclear cells (PBMCs)

For human subjects, venous blood was collected from each individual into sodium heparin tubes and processed within 2 h after sampling. Histopaque-1077 (Sigma–Aldrich, Cat#10771) or Human Lymphocyte Isolation Solution (Dakewe, Cat#7111011) density gradient centrifugation was performed to isolate PBMCs from whole blood through a 25-min centrifugation in 15 mL centrifuge tube (BDBIO, Cat#H806001) at 800 rcf. For mice, 250 μL of peripheral blood was collected from the retro-orbital sinus and transferred into Eppendorf tubes containing EDTA after anaesthesia induction with a continuous flow of 3% isoflurane. PBMCs were then isolated using Histopaque-1083 (Sigma–Aldrich, Cat#10831) according to the manufacturer’s instructions.

### Clinical parameters assays

Glycated haemoglobin (HbA1c), C-peptide, glycated albumin (GA) and serum lipid assays referred to our previously published articles.^12^^,^^20^ We measured islet autoantibodies by radioligand assays, namely, glutamic acid decarboxylase antibody (GADA), protein tyrosine phosphatase autoantibody (IA-2A), and zinc transporter 8 autoantibody (ZnT8A). The positive thresholds for antibodies were as follows: GADA ≥ 0.05 titres, IA-2A ≥ 0.02 titres, and ZnT8A ≥ 0.011 titres. Our laboratory achieved 82% sensitivity and 96.7% specificity for GADA, 76% sensitivity and 100% specificity for IA-2A, and 76% sensitivity and 100% specificity for ZnT8A in the islet autoantibody standardisation programme (IASP 2020).

### Experimental animals

NOD mice (female, aged 4, 8, 10, and 12 weeks) were purchased from HFK Biosciences (Beijing, China). For the induction of diabetes with cyclophosphamide (Sigma–Aldrich, Cat#C0768), NOD mice treated with a single injection of cyclophosphamide (200 mg/kg, intraperitoneally [i.p.]) at indicated time. To investigate the preventive role of anti-CD132 in T1D, the monoclonal anti-CD132 (common γ chain) antibody 3E12 (BioXCell, Cat#BE0271, RRID:AB_2687794) and the anti-IgG2b isotype control (BioXCell, Cat#BE0090, RRID:AB_1107780) were administered injection (10 mg/kg, i.p.) on days 0, 2, 4, 6, 8, 10, 12, and 14. To investigate the preventive role of anti-IL-15 in T1D, the monoclonal anti-IL-15 antibody AIO.3 (BioXCell, Cat#BE0315, RRID:AB_2754553) and the anti-IgG2a isotype control (BioXCell, Cat#BE0089, RRID:AB_1107769) were administered injection (25 μg per mouse, i.p.) on days 0, 2, 4, 6, 8, 10, 12, and 14. Blood glucose levels were recorded daily for up to 28 days. If blood glucose levels consistently exceeded 13.9 mmol/L over two successive days, the onset of diabetes was indicated. To elucidate the therapeutic role of anti-CD132 in T1D, the mice were randomised into four treatment groups after diabetes onset: 1) Control group: IgG f(ab’)2 fragment isotype control plus anti-IgG2b isotype control; 2) Anti-CD3 group: anti-CD3 plus anti-IgG2b isotype control; 3) Anti-CD132 group: anti-CD132 plus IgG f(ab’)2 fragment isotype control; and 4) Anti-CD3 and anti-CD132 group: anti-CD3 plus anti-CD132. Anti-mouse CD3ε F(ab’)2 fragment (BioXCell, Cat#BE0001-1FAB, RRID:AB_2687679) and IgG f(ab’)2 fragment isotype control (BioXCell, Cat#BE0091-FAB, RRID:AB_2687680) were administered 50 μg per mouse i.p., daily for 5 days.^21^ Anti-CD132 and anti-IgG2b isotype control were administered 10 mg/kg per mouse i.p., daily for 5 days. Blood glucose was measured every day for up to 35 days, and values less than 13.9 mmol/L indicated remission. The pancreatic tissues were treated with 4% paraformaldehyde for fixation. Insulitis severity was assessed via haematoxylin and eosin (H&E) staining and was scored as follows: 0 for no infiltration, 1 for less than 25% islets infiltration, 2 for 25–50% islets infiltration, and 3 for more than 50% islets infiltration.

C57BL/6 mice were purchased from GemPharmatech Co., Ltd. T1D was induced in 8-week-old male C57BL/6 mice by multiple low-dose injections of streptozotocin (STZ, Sigma–Aldrich, Cat#S0130). STZ was dissolved in fresh cold 0.01 M citrate buffer (pH 4.5). After fasting for 4–6 h, the mice were i.p., injected with STZ solution at a dose of 40 mg/kg for five consecutive days. All animals were freely provided with water and food in a specific pathogen-free (SPF) animal facility with constant temperature and humidity, and subjected to a light–dark cycle for 12 h. The animal use protocol was reviewed and approved by the Institutional Animal Care and Use Committee (IACUC), The Second Xiangya Hospital, Central South University, China.

### Flow cytometry

First, we prepared single-cell suspensions from humans and mice. For surface staining, PBMCs, spleen, and pancreatic lymph node (PLN) cells were performed in Hank’s balanced salt solution (HBSS, Solarbio, Cat#H1045) with 0.5% bovine serum albumin (BSA, Sigma–Aldrich, Cat#A8806) for 30 min at 4 °C. For intracellular cytokine staining, the cells were stimulated with leucocyte activation cocktail (BD, Cat#550583, RRID:AB_2868893) for 5 h in a 37 °C incubator and then permeabilized with Intracellular Fixation & Permeabilization Buffer Set (eBioscience, Cat#88-8824). For Ki67 staining, Transcription Factor Buffer Set (BD, Cat#562574, RRID:AB_2869424) was used. For glucose uptake detection, the cells were performed in glucose-free Roswell Park Memorial Institute (RPMI) 1640 medium with 100 μM 2-NBDG (2-deoxy-2-[(7-nitro-2,1,3-benzoxadiazol-4-yl)amino]-D-glucose, Cayman, Cat#11046) for 30 min in a 37 °C incubator. For fatty acid (FA) uptake detection, the cells were performed in FA-free RPMI 1640 medium with 1 μM BODIPY FL C16 (4,4-difluoro-5,7-dimethyl-4-bora-3a,4a-diaza-s-indacene-3-hexadecanoic acid, Invitrogen, Cat#D3821) for 30 min in a 37 °C incubator. For mitochondrial mass detection, the cells were performed in HBSS with 100 nM MitoTracker® Deep Red FM (Invitrogen, Cat#M22426) for 30 min in a 37 °C incubator. Surface staining was performed after terminating metabolite uptake by adding fourfold ice-cold phosphate-buffered saline (PBS, Gibco, Cat#C10010500BT; Biosharp, Cat#BL302A; EallBio, Cat#03.15018C). All fluorescent-labelled anti-human and anti-mouse monoclonal antibodies utilised in the study were shown in the supplementary materials (Supplementary Table S1). The antibodies selected for this study have been extensively validated in previous publications. We verified their specificity using isotype controls and fluorescence minus one (FMO) controls as negative controls, alongside appropriate positive controls. Additionally, we titrated multiple antibody concentrations to determine the optimal staining condition. All experimental results were replicated in at least three independent experiments. Flow cytometric data were collected on a NovoCyte Quanteon™ (Agilent) or Northern Lights NL-3000 (CYTEK). The analysis was conducted with FlowJo version 10.8.1 software. The gating strategy for B cell subsets was shown in the supplementary figure (Supplementary Fig. S2a).

### Cell purification and sorting

Human B cells and NOD mouse splenic B cells were enriched with MojoSort™ Human Pan B Cell Isolation Kit (BioLegend, Cat#480082) and MojoSort™ Mouse Pan B Cell Isolation Kit II (BioLegend, Cat#480088). The sorting purity was > 95.0%. The isolated B cells were stained with anti-CD19/B220 and anti-CD226 antibodies for 30 min on ice. B cells with positive and negative expression of CD226 were sorted via fluorescence-activated cell sorting (FACS) with BD FACS Aria IIu instrument. Naive CD4^+^ T cells were purified using Naive CD4^+^ T Cell Isolation Kit (Miltenyi, Cat#130-104-453) according to the manufacturer’s instructions. CD14^+^ monocytes and CD8^+^ T cells were sorted via FACS with BD FACS Aria IIu instrument.

### Extracellular acidification rate (ECAR) assay

We measured real-time ECAR of purified CD226^+^ B cells and CD226^−^ B cells from T1D patients using the Glycolytic Stress Test Kit (Seahorse Bioscience, Cat#103020-100) according to the manufacturer’s protocol by the Seahorse Extracellular Flux analyser (Seahorse Bioscience). Briefly, sorted cells were seeded in a 96-well plate coated with Cell-Tak (Corning, Cat#354240). Data were normalised to cell number. During the ECAR measurement, Glucose (10 mM), Oligomycin A (1 μM), and 2-DG (50 mM) were added to the cells. Data were analysed with Wave software version 2.6 (Agilent Technologies).

### RNA-seq and quantitative reverse transcriptase polymerase chain reaction (qRT-PCR)

First, 2 × 10^5^ CD226^+^ and CD226^−^ B cells from NOD mice spleen were sorted. The Eastep™ Super Total RNA Extraction Kit (Promega, Cat#LS1040) or Trizol Total RNA Extraction Kit (Absin, Cat#abs9331) was subsequently used to extract RNA from the sorted cells. Next, RNA sequencing was conducted at the Beijing Genomics Institution (BGI). The data have been uploaded to the National Centre for Biotechnology Information (NCBI) and are accessible through BioProject ID PRJNA1116527 (available at https://dataview.ncbi.nlm.nih.gov/object/PRJNA1116527?reviewer=q0o808npbgqott2hgieqh9tng9). The RNA quality, including mRNA concentration, RIN value, and 28S/18S, was examined with a Fragment Analyser. All samples met the required quality standards for sequencing, with RIN values exceeding 7.0 and clear 28S/18S peaks, ensuring high-quality RNA for downstream analysis. RNA sequencing was performed on the Illumina NovaSeq 6000 platform with paired-end 150 bp reads. Raw reads were processed to generate clean reads by removing adaptor sequences, filtering low-quality reads (Q-score < 20), and discarding reads with ambiguous bases. Each sample yielded at least 30 million clean reads. The read counts were expressed as fragments per kilobase of exon per million fragments mapped (FPKM). We used Bowtie2 (v2.2.5) and RSEM software package (v1.2.8) to align and quantify RNA-seq reads. Differential gene analysis was carried out using the ‘DEseq2’ method. Genes showing at least a two-fold upregulation or downregulation with a false discovery rate (FDR) adjusted P value < 0.05 were considered as differential express genes (DEGs). The DEGs were annotated by Gene Ontology (GO) and Kyoto Encyclopedia of Genes and Genomes (KEGG) functional analyses. In addition, we used gene set enrichment analysis (GSEA) for biological functional enrichment analysis.

The RNA was reverse-transcribed into cDNA via the PrimeScript RT Master Mix (TAKARA, Cat#RR036A) or Hifair III 1st Strand cDNA Synthesis SuperMix for qPCR (Yeasen, Cat#11141ES60). Quantitative PCR was carried out with GoTaq qPCR Master Mix (Promega, Cat#A6001) on ABI QuantStudio 6 Flex (Applied Biosystems). Primer sequencing performed in the research were provided in supplementary table (Supplementary Table S2).

### Predicting cytokines upstream of CD226

Cytokine Signalling Analyser (CytoSig, https://cytosig.ccr.cancer.gov) is a data-driven infrastructure that enables systematic investigation of cellular responses to cytokines. It provides both databases (NCBI GEO and ArrayExpress) of cytokine-modulated target genes and predictive models of cytokine signalling cascades derived from transcriptomic profiles.^22^ We utilised the CytoSig to predict upstream cytokines regulating CD226 by inputting CD226 as the target gene. We set the analysis thresholds for a minimum number of entries (minimum count) in each group for three and P-value cutoff for 0.05.

### Cell culture in vitro

We cultured PBMCs from T1D donors for 2 days using monoclonal anti-human CD226 antibody (10 μg/mL; R&D Systems, Cat#MAB666, RRID:AB_2072624) or mouse IgG1 isotype control (10 μg/mL; R&D Systems, Cat#MAB002, RRID:AB_357344) to inhibit CD226 on B cells in vitro. To assess the responsiveness of human B cells to interleukin-21 (IL-21), IL-15, and interleukin-4 (IL-4), the cells were cultured for 2 days with IL-21 (20 ng/ml; Peprotech, Cat#200-21), IL-15R alpha & IL-15 (20 ng/ml; MedChemExpress, Cat#HY-P77699), or IL-4 (20 ng/ml; Peprotech, Cat#200-04). After the incubation period, the cells were stained to identify surface markers and intracellular cytokines. Additional experiments included treating the cells with QNZ (20 nM; Selleck, Cat#545380-34-5) or BAY 11-7082 (10 μM; MedChemExpress, Cat#HY-13453), specific inhibitors of NF-κB. The purified monocytes were stimulated with 10 ng/ml lipopolysaccharides (LPS, Sigma–Aldrich, Cat#L2880) for 24 h. The purified CD8^+^ T cells were stimulated with anti-human CD3 (5 μg/ml; BioLegend, Cat#317326, RRID:AB_11150592) and anti-human CD28 (2 μg/ml; BioLegend, Cat#302934, RRID:AB_11148949) for 24 h.

### T cell-B cell co-cultures

We stimulated NOD mouse splenic B cells with IL-15R alpha & IL-15 (20 ng/ml; MedChemExpress, Cat#HY-P78558) in vitro for 2 days. Purified naive CD4^+^ T cells were stimulated with anti-CD3 (5 μg/ml; BD, Cat#553057, RRID:AB_394590) and anti-CD28 (2.5 μg/ml; BD, Cat#553294, RRID:AB_394763) and maintained in a 1640 medium supplemented with IL-2 (10 ng/ml; Peprotech, Cat#212-12). IL-15-primed B cells or B cells were co-cultured with naive CD4^+^ T cells at a ratio of 1:5. Naive CD4^+^ T cells were cultured supplemented with IL-12 (10 ng/ml; Peprotech, Cat#210-12) for Th1 generation or IL-6 (20 ng/ml; Peprotech, Cat#216-16), TGF-β (5 ng/ml; Peprotech, Cat#100-21), IL-23 (10 ng/ml; Peprotech, Cat#200-23) for Th17 generation. Flow cytometry analysis was performed after 4 days of culture.

### Enzyme-linked immunosorbent assay (ELISA)

TNF-α and IFN-γ secretion in supernatants of human CD226^+^ B cell and CD226^−^ B cell cultures after 48-h LPS (5 μg/ml; Sigma–Aldrich, Cat#L2880) stimulation were measured using the Human Tumour necrosis factor α ELISA Kit (CUSABIO, Cat#CSB-E04740h, https://www.cusabio.com/) and the Human Interferon Gamma ELISA Kit (JONLNBIO, Cat#JL12152-96T), respectively. We detected the serum IL-15 secretion level with the Human IL-15 Quantikine ELISA Kit (R&D Systems, Cat#D1500). IL-15 secretion in supernatants of human monocyte and CD8^+^ T cell cultures after 24 h was measured using the Human Interleukin 15 ELISA Kit (Wuhan Fine Biotech Co., Ltd., Cat#EH0177) according to the manufacturer’s instructions.

### Statistical analysis

We performed the statistical analyses with IBM SPSS 26.0 and GraphPad Prism 9.0. Normality tests were conducted using either the Kolmogorov–Smirnov test (KS) or the Shapiro–Wilk test (SW). Data following a normal distribution was typically represented as the mean ± standard error of the mean (SEM), while data deviating from a normal distribution was displayed as the median (25th-75th percentile). We performed Student’s t-test to compare two groups that were normally distributed, whereas the Mann–Whitney U test to compare two groups that deviated from a normal distribution. The treatments of cells from the same patient were performed by paired t-tests, and the robustness of the findings was confirmed using the Wilcoxon signed-rank test. We performed one-way analysis of variance (ANOVA) or the Kruskal–Wallis test followed by Bonferroni’s correction or Tukey’s multiple comparisons test adjustments for comparisons between more than two groups. We used the Chi-square test or Fisher’s statistical measure to compare qualitative data. For correlation analyses, Pearson correlation analysis was performed for normally distributed data, whereas Spearman correlation analysis was performed for data deviating from normal distribution. Survival curves indicating diabetes incidence were analysed by log-rank test. P values below 0.05 were deemed to be significant differences.

### Role of funders

The funders were not involved in the study design, data collection, data analysis, interpretation, or the writing of the report.

## Results

### The proportion of CD226^+^ B cells is elevated and correlates with disease progression in T1D patients

**Fig. 1:**
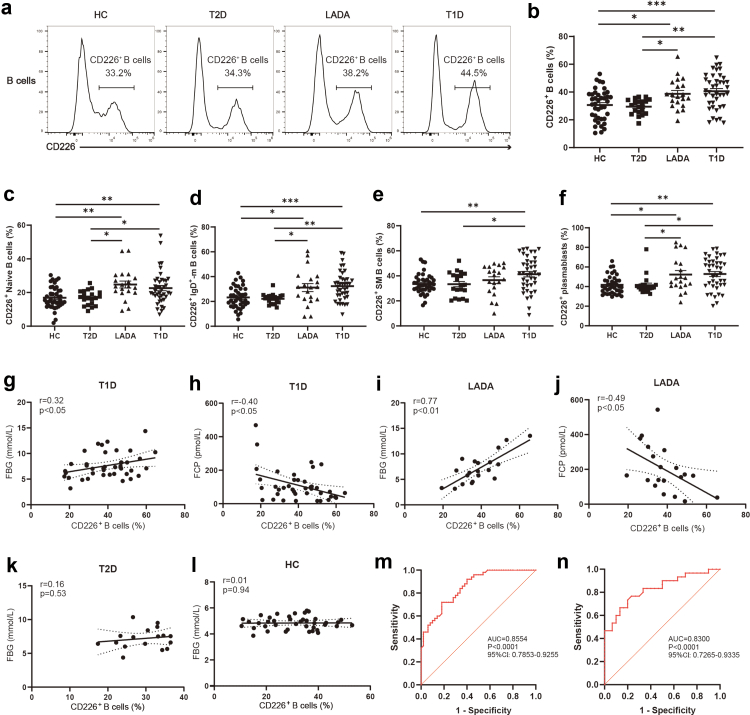
**The proportion of CD226^+^ B cells is correlated with disease progression in T1D patients.** (a and b) Representative flow cytometry plots (a) and columnar scatter plots (b) displaying the expression of CD226 on B cells in HC (n = 40), T2D (n = 20), LADA (n = 20), and T1D (n = 40). One-way analysis of variance (ANOVA) followed by adjustments was used for multiple comparisons. Each point represents an individual. Horizontal bars represent the mean ± SEM. (c–f) Columnar scatter plots displaying the expression of CD226 on naive B cells (c), IgD^+^-memory B cells (d), switched memory (SM) B cells (e), and plasmablasts (f) in HC (n = 40), T2D (n = 20), LADA (n = 20), and T1D (n = 40), as assessed by flow cytometric analysis. One-way ANOVA followed by adjustments was used for multiple comparisons. Each point represents an individual. Horizontal bars represent the mean ± SEM. (g–j) Relationships between CD226^+^ B cells and clinical parameters from T1D (n = 40) and LADA (n = 20). Correlations between the percentage of CD226^+^ B cells and FBG (g), FCP (h) in T1D patients. Correlations between the percentage of CD226^+^ B cells and FBG (i), FCP (j) in LADA patients. (k and l) Relationships between CD226^+^ B cells and clinical parameters from T2D (n = 20) and HC (n = 40). Correlations between the percentage of CD226^+^ B cells and FBG in T2D (k) patients and HC (l). Pearson or Spearman’s rank correlation was used for correlation analyses. Linear regression is shown with 95% CIs (dotted area). (m and n) ROC curves for CD226^+^ B cells were used to predict T1D (m) and LADA (n). ∗P < 0.05. ∗∗P < 0.01. ∗∗∗P < 0.001. Abbreviations: HC, healthy controls; T2D, type 2 diabetes; LADA, latent autoimmune diabetes in adults; T1D, type 1 diabetes; SEM, standard error of the mean; IgD^+^-m B cells, IgD^+^-memory B cells; SM B cells, switched memory B cells; FBG, fasting blood glucose; FCP, fasting C-peptide; ROC, receiver operating characteristic curve; AUC, area under the curve.

To observe the characteristics of CD226 in T1D patients, we performed flow cytometry to assess CD226 expression in B cells from forty HC, forty T1D patients, twenty T2D patients, and twenty LADA patients (Table 1). We found a significant increase in the frequency of CD226^+^ B cells in T1D patients compared with HC. Similarly, the proportion of CD226^+^ B cells was greater in LADA patients than in T2D patients (Fig. 1a and b). Interestingly, there was no difference between T2D patients and HC. To further understand whether the increased proportion of CD226^+^ cells in specific B lymphocyte subsets, we analysed CD226 expression in four subsets: naive B cells, IgD^+^-memory B cells, switched memory (SM) B cells, and plasmablasts. We found that the proportion of CD226^+^ cells was greater in assayed B lymphocyte subpopulations from T1D patients than in those from HC (Fig. 1c–f). Furthermore, we observed that the frequency of CD226 expression was higher in functional B cell subsets such as plasmablasts than in immature B cell subsets like naive B cells, indicating that a higher percentage of the CD226^+^ B cell subset seems more active functionally.

Next, we proceeded to determine whether the alterations in CD226^+^ B cells are related to the clinical features of T1D patients. The frequency of CD226^+^ B cells was positively related to FBG (r = 0.32, P < 0.05), HbA1c (r = 0.32, P < 0.05) and GA levels (r = 0.37, P < 0.05), and negatively related to FCP levels (r = −0.40, P < 0.05) in T1D patients. The frequency of CD226^+^ B cells was positively related to FBG (r = 0.77, P < 0.01), HbA1c (r = 0.71, P < 0.01) and GA levels (r = 0.49, P < 0.05), and negatively related to FCP levels (r = −0.49, P < 0.05) in LADA patients (Fig. 1g–j; Supplementary Fig. S1a–d). The proportion of CD226^+^ B cells is independent of FBG levels in T2D patients or HC (Fig. 1k and l), suggesting that the elevated frequency of CD226^+^ B cells is mediated primarily by immune factors rather than hyperglycemia. We further stratified T1D patients into subgroups on the basis of their FBG, HbA1c, and FCP levels for analysis. We found that T1D patients with poorer blood glucose control (FBG > 7.1 mmol/L, HbA1c > 7.5%) and pancreatic islet function (FCP ≤ 90.0 pmol/L) had a higher proportion of CD226^+^ B cells (Supplementary Fig. S1e–g). Similarly, LADA patients with poorer blood glucose control (FBG > 6.7 mmol/L, HbA1c > 7.0%) and pancreatic islet function (FCP ≤ 165.0 pmol/L) had a higher proportion of CD226^+^ B cells (Supplementary Fig. S1h–j). Next, we analysed the correlation between CD226^+^ B cells and the three islet autoantibodies. There was uniformity in the proportion of CD226^+^ B cells among the T1D and LADA patients with one, two, or three positive autoantibodies (Supplementary Fig. S1k and m). Furthermore, the frequency of CD226^+^ B cells was similar among T1D patients regardless of whether they had positive or negative results for GADA, IA-2A, or ZnT8A (Supplementary Fig. S1l). Interestingly, the proportion of CD226^+^ B cells was greater in GADA^+^ LADA patients than in GADA^−^ LADA patients, which may be influenced by the small sample size (Supplementary Fig. S1n). Receiver operating characteristic (ROC) curve analysis revealed that the frequency of CD226^+^ B cells assisted in T1D diagnosis. Detection of peripheral blood CD226^+^ B cell proportions from individuals distinguished between T1D patients and HC (sensitivity of 72.0%, specificity of 82.0%) (Fig. 1m), and between LADA patients and T2D patients (sensitivity of 76.7%, specificity of 76.7%) (Fig. 1n).

### Increased activation and production of pro-inflammatory cytokines in CD226^+^ B cells from T1D patients

**Fig. 2:**
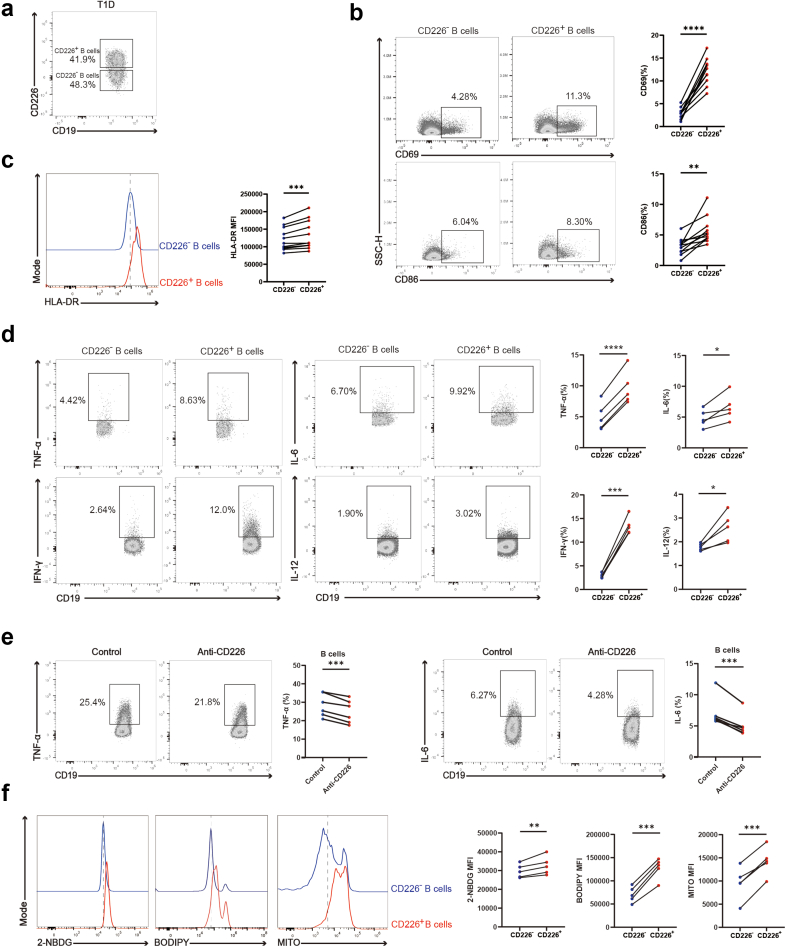
**Functional and metabolic analysis of CD226^+^ B cells and CD226^−^ B cells in T1D patients.** (a) Peripheral blood B cells were categorised into CD226^+^ and CD226^−^ B cells on the basis of CD226 expression in T1D. (b–d) Representative flow cytometry plots and scatter plots for paired t-tests of CD69 (n = 11), CD86 (n = 11) (b), HLA-DR (n = 11) (c), TNF-α, IFN-γ, IL-6, and IL-12 (n = 5) (d) expression in CD226^+^ and CD226^−^ B cells from T1D patients. (e) Representative flow cytometry plots and scatter plots for paired t-tests of TNF-α and IL-6 expression in B cells between control group and anti-CD226 group (n = 6). (f) Representative flow cytometry plots and scatter plots for paired t-tests of 2-NBDG, BODIPY, and MITO expression in CD226^+^ and CD226^−^ B cells from T1D patients (n = 5). ∗P < 0.05. ∗∗P < 0.01. ∗∗∗P < 0.001. ∗∗∗∗P < 0.0001. Abbreviations: T1D, type 1 diabetes; SSC-H, side scatter height; 2-NBDG, 2-deoxy-2-[(7-nitro-2,1,3-benzoxadiazol-4-yl)amino]-D-glucose; BODIPY, boron-dipyrromethene; MITO, MitoTracker® Deep Red FM.

To assess the function of CD226^+^ B cell subset in T1D, we isolated PBMCs from T1D patients. B cells were categorised into CD226^+^ and CD226^−^ B cells on the basis of CD226 expression in T1D (Fig. 2a) and LADA patients (Supplementary Fig. S2b). We found that CD226^+^ B cells presented increased activation and antigen presentation markers such as CD69, CD86, and HLA-DR (Fig. 2b and c), a trend that was also present in HC (Supplementary Fig. S2c). CD226^+^ B cells also presented enhanced pro-inflammatory cytokine production like TNF-α, IFN-γ, IL-6, and IL-12 than those in CD226^−^ B cells from T1D (Fig. 2d) and LADA patients (Supplementary Fig. S2e). ELISA revealed that CD226^+^ B cells in T1D secreted more pro-inflammatory cytokines, including TNF-α and IFN-γ, compared to CD226^−^ B cells (Supplementary Fig. S2d). Subsequently, we observed that in vitro inhibition of CD226 in PBMCs from T1D donors led to a reduction in the production of pro-inflammatory cytokines by B cells (Fig. 2e) These results suggest that the activation and pro-inflammatory functions of CD226^+^ B cells may aggravate T1D. We further investigated the metabolic phenotype, given the close association between B cell metabolism and function. We observed higher intracellular glucose uptake, fatty acid uptake, and mitochondrial mass in CD226^+^ B cells than in CD226^−^ B cells in T1D patients, which proved by elevated levels of 2-NBDG, BODIPY and MitoTracker, respectively (Fig. 2f). In addition, we found that CD226^+^ B cells had increased glycolysis activity compared to CD226^−^ B cells in T1D patients by their ECAR measurement (Supplementary Fig. S2f–i).

### Increased activation, co-stimulation, proliferation, and pro-inflammatory responses in CD226^+^ B cells from NOD mice

**Fig. 3:**
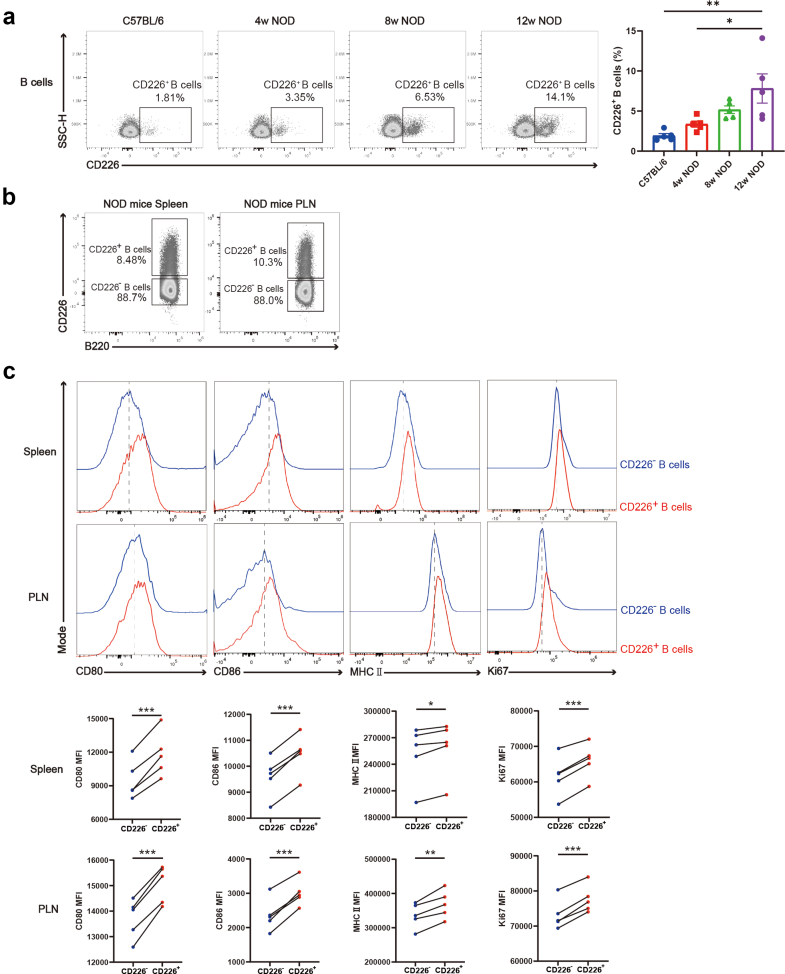
**Functional analysis of CD226^+^ B cells and CD226^−^ B cells in NOD mice.** (a) The expression levels of CD226 on B cells in PLN at 4, 8, and 12 weeks of age in NOD mice and 10–12 weeks of age in C57BL/6 mice (n = 5). One-way ANOVA followed by adjustments was used for multiple comparisons. (b) B cells were categorised into CD226^+^ and CD226^−^ B cells on the basis of CD226 expression in the spleen and PLN of NOD mice. (c) Representative flow cytometry plots and scatter plots for paired t-tests of CD80, CD86, MHC II, and Ki67 expression in CD226^+^ and CD226^−^ B cells from the spleen and PLN of NOD mice (n = 5). ∗P < 0.05. ∗∗P < 0.01. ∗∗∗P < 0.001. Abbreviations: NOD, non-obese diabetic; SSC-H, side scatter height; PLN, pancreatic lymph node.

To further characterise the pathogenic effect of CD226^+^ B cells in T1D, we utilised NOD mice for studies. We discovered that the proportion of CD226^+^ B cells in PLN was greater in NOD mice than in C57BL/6 mice. As NOD mice aged with disease progression, the frequency of CD226^+^ B cells in PLN gradually increased (Fig. 3a), with a consistent trend observed in peripheral blood (Supplementary Fig. S3a). Furthermore, we categorised B cells from the spleen and PLN of 10-week-old prediabetic NOD mice into CD226^+^ and CD226^−^ B cells on the basis of CD226 expression (Fig. 3b). Compared with CD226^−^ B cells, CD226^+^ B cells exhibited increased expression levels of CD80, CD86, MHC II, and Ki67, indicating enhanced activation, antigen presentation, and proliferation capacities (Fig. 3c). Consistent with results in humans, CD226^+^ B cells from NOD mice produced more pro-inflammatory cytokines (Supplementary Fig. S3b), suggesting that they are potentially involved in the development of T1D. Additionally, CD226^+^ B cells presented increased uptake of glucose and fatty acids (Supplementary Fig. S3c).

### The NF-κB signalling pathway promotes the generation and pro-inflammatory responses of CD226^+^ B cells

**Fig. 4:**
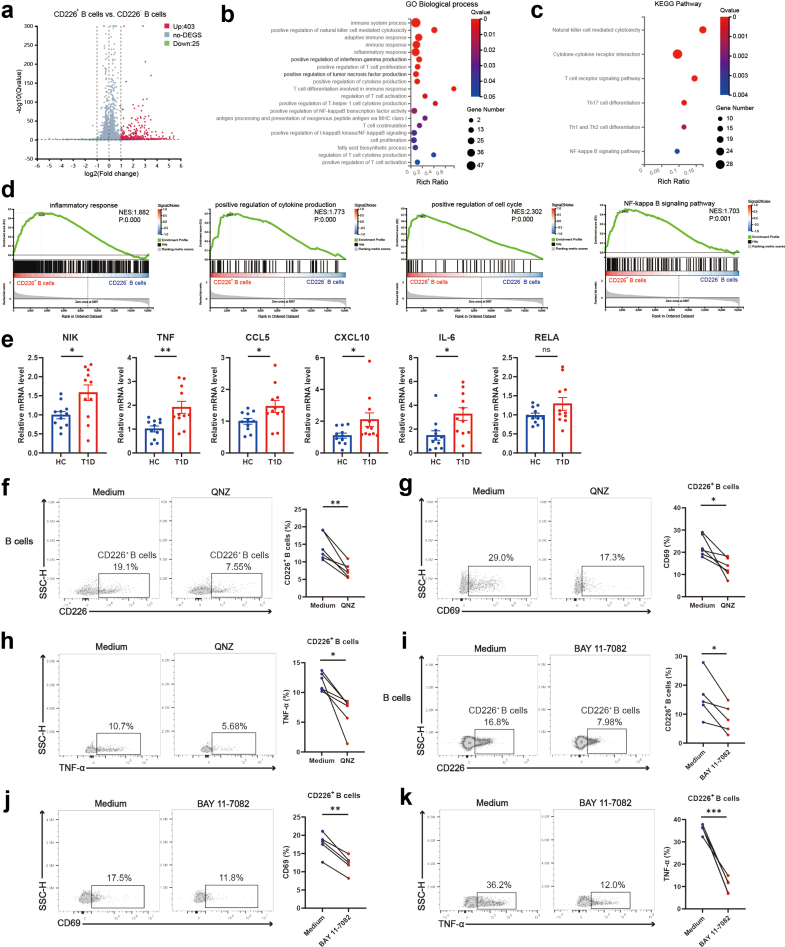
**NF-κB signalling drives the heightened inflammatory response in CD226^+^ B cells.** (a) Volcano plot illustrating the differential expression of genes between CD226^+^ B cells and CD226^−^ B cells in NOD mice (n = 5). The adjusted P value of each gene was determined by DESeq2 with Benjamini-Hochberg false discovery rate (FDR) correction. Genes meeting the criteria of adjusted P < 0.05 and fold change > 2.0 were considered significant. (b and c) Functional annotation analysis of DEGs using GO (b) and KEGG (c) analyses. Statistical disparities between enriched terms and pathways were adjusted utilising Bonferroni’s test, applying FDR adjusted P-value ≤ 0.05 to ensure accuracy. (d) GSEA for inflammatory response, positive regulation of cytokine production, positive regulation of cell cycle and NF-kappa B signalling pathway associated genes in CD226^+^ B cells versus CD226^−^ B cells. (e) Quantitative real-time PCR (qPCR) analysis for NF-κB target genes expression in B cells from T1D patients and HC (n = 11). Student’s t-test was used for comparing two groups. (f) Representative flow cytometry plots and scatter plots for paired t-tests demonstrating the inhibition of CD226 expression in B cells upon QNZ treatment (n = 6). (g and h) Representative flow cytometry plots and scatter plots for paired t-tests demonstrating the inhibition of activation (g) and inflammatory cytokine production (h) in CD226^+^ B cells upon QNZ treatment (n = 6). (i) Representative flow cytometry plots and scatter plots for paired t-tests demonstrating the inhibition of CD226 expression in B cells upon BAY 11-7082 treatment (n = 5). (j and k) Representative flow cytometry plots and scatter plots for paired t-tests demonstrating the inhibition of activation (j) and inflammatory cytokine production (k) in CD226^+^ B cells upon BAY 11-7082 treatment (n = 5). ∗P < 0.05. ∗∗P < 0.01. ∗∗∗P < 0.001. Abbreviations: DEGs, differential express genes; GO, Gene Ontology; KEGG, Kyoto Encyclopedia of Genes and Genomes; GSEA, gene set enrichment analysis; NES, normalised enrichment score; HC, healthy controls; T1D, type 1 diabetes; ns, not significant; SSC-H, side scatter height.

To delve deeper into the regulatory mechanisms of the aforementioned results, we conducted RNA-seq technology to clarify the transcriptomes of CD226^+^ (n = 5) and CD226^−^ B cells (n = 5) from prediabetic NOD mice. We identified 403 upregulated genes and 25 downregulated genes as differential express genes (DEGs) [FDR-adjusted P < 0.05, log_2_ (fold change) > 1] (Fig. 4a). Gene Ontology (GO) analysis revealed enrichment of the following terms in CD226^+^ B cells compared with CD226^−^ B cells: “positive regulation of natural killer cell mediated cytotoxicity”, “inflammatory response”, “positive regulation of interferon-gamma production”, “positive regulation of cytokine production”, “positive regulation of T cell proliferation”, “positive regulation of NF-kappaB transcription factor activity”, and “T cell costimulation” (Fig. 4b). Kyoto Encyclopedia of Genes and Genomes (KEGG) analysis also demonstrated enrichment of the “NF-kappa B signalling pathway” in CD226^+^ B cells (Fig. 4c). Gene set enrichment analysis (GSEA) revealed that CD226^+^ B cells presented increased inflammatory cytokine production and proliferation capacity compared with CD226^−^ B cells (Fig. 4d; Supplementary Fig. S4a). Notably, both GO and GSEA revealed enrichment of lipid synthesis and metabolism in CD226^+^ B cells (Fig. 4b; Supplementary Fig. S4a), which is in agreement with our previous results on fatty acid uptake. Importantly, the results of the GO/KEGG/GSEA analyses all pointed to the involvement of the NF-κB signalling pathway in the function of CD226^+^ B cells (Fig. 4b–d). To validate this, we found that the mRNA expression levels of NF-κB pathway target genes in CD226^+^ B cells were higher than those in CD226^−^ B cells from NOD mice (Supplementary Fig. S4b). Furthermore, we assessed the mRNA expression levels of NF-κB signalling pathway-related genes in circulating B cells isolated from T1D patients and HC. The expression of NIK, TNF, CCL5, CXCL10, and IL-6 was elevated in T1D patients compared with HC (Fig. 4e). Moreover, treatment of patient-derived B cells with QNZ or BAY 11-7082 (both the inhibitor of NF-κB) inhibited the expression of CD226 and the activation and pro-inflammatory cytokine production of CD226^+^ B cells (Fig. 4f–k).

### IL-15 enhances the generation and activation of CD226^+^ B cells in vitro

**Fig. 5:**
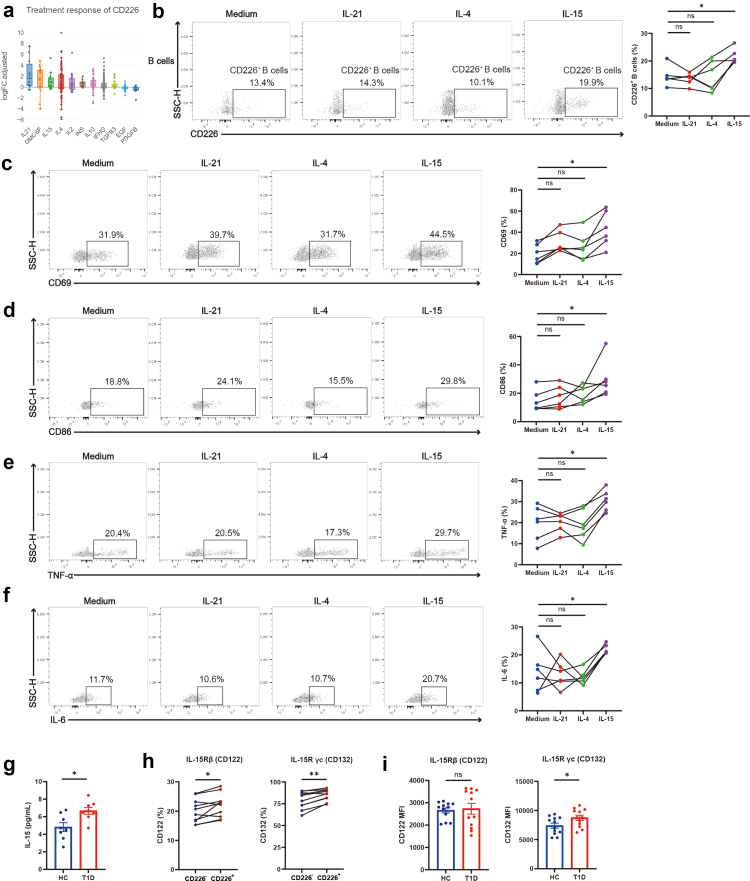
**IL-15 promotes the generation and activation of CD226^+^ B cells.** (a) Forecasting the regulator of the CD226 gene. (b) Representative flow cytometry plots and line charts of CD226 expression on B cells isolated from T1D patients following 48-h culture with either medium control, 20 ng/ml IL-21, 20 ng/ml IL-4, or 20 ng/ml IL-15R alpha & IL-15 (n = 6). One-way repeated measures ANOVA followed by adjustments was used for multiple comparisons. Lines connect the same sample. (c–f) Representative flow cytometry plots and line charts of CD69 (c), CD86 (d), TNF-α (e), and IL-6 (f) expression in CD226^+^ B cells from T1D patients after 48-h indicated stimulation (n = 6). One-way repeated measures ANOVA followed by adjustments was used for multiple comparisons. Lines connect the same sample. (g) The levels of IL-15 in the serum of HC and T1D patients (n = 8). Student’s t-test was used for comparing two groups. (h) The expression of IL-15R (CD122 and CD132) was compared between CD226^+^ B cells and CD226^−^ B cells in T1D patients (n = 10). Paired t-test was used for comparing two groups. (i) The expression of IL-15R (CD122 and CD132) was compared between HC and T1D patient B cells (n = 12). Student’s t-test was used for comparing two groups. ∗P < 0.05. ∗∗P < 0.01. Abbreviations: SSC-H, side scatter height; HC, healthy controls; T1D, type 1 diabetes.

We conducted additional research into the factors influencing CD226^+^ B cells, with particular emphasis on cytokines. According to predictions from the CytoSig database, IL-15 was identified as a potential regulator of the CD226 gene (Fig. 5a). In-vitro IL-15 treatment of T1D donor B cells led to elevated levels of CD226^+^ B cells (Fig. 5b), along with increased expression of CD69, CD86, TNF-α, and IL-6 (Fig. 5c–f) by CD226^+^ B cells. Because IL-21 and IL-4 are predicted to be upstream cytokines of CD226, we compared the inductive effects of IL-15 on CD226^+^ B cells with those of IL-21 and IL-4, revealing only IL-15 as an effective inducer (Fig. 5b–f). To investigate the ability of IL-15-primed B cells on autoreactive T cell activation, we isolated naive CD4^+^ T cells and co-cultured them with IL-15-primed B cells or B cells. We found that IL-15-primed B cells significantly enhanced the differentiation of naive CD4^+^ T cells into T helper type 1 (Th1) and T helper type 17 (Th17) subsets, as evidenced by increased production of IFN-γ and IL-17, respectively, compared to B cells (Supplementary Fig. S5a). Furthermore, we observed increased levels of IL-15 in the serum from T1D patients compared with HC (Fig. 5g). IL-15Rα (Interleukin-15 receptor α, CD215) expressed at lower levels in T1D B cells (Supplementary Fig. S5b), therefore we focused on the other two receptors, IL-15Rβ (Interleukin-15 receptor β, CD122) and IL-15R γc (Interleukin-15 receptor common γ chain, CD132). They were both higher in CD226^+^ B cells than in CD226^−^ B cells from T1D patients (Fig. 5h). The expression of IL-15R γc (CD132) on B cells was significantly greater in T1D patients than in HC (Fig. 5i).

### Monocyte/macrophage-derived IL-15 promotes the generation and activation of CD226^+^ B cells

**Fig. 6:**
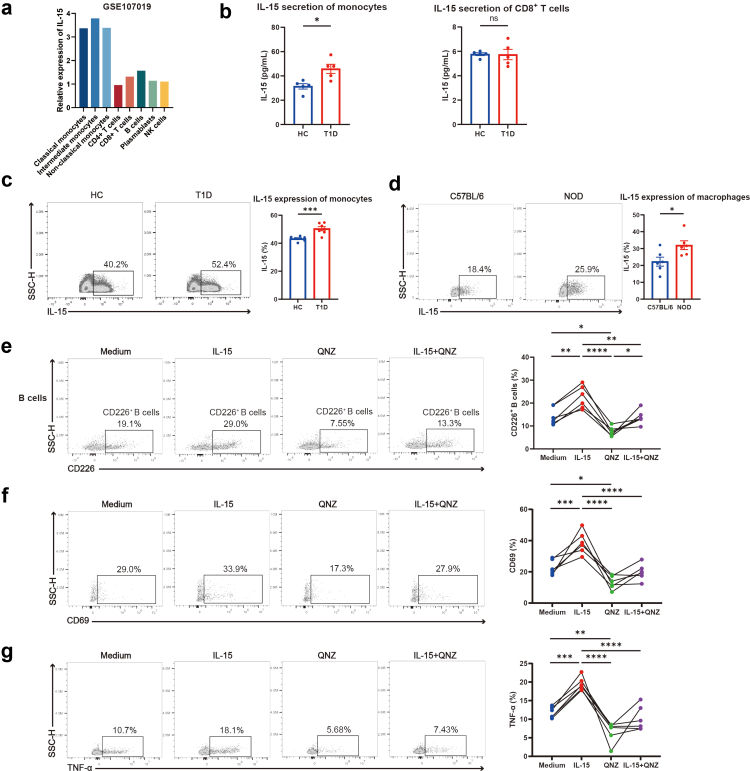
**Monocyte/macrophage-derived IL-15 promotes the generation and activation of CD226^+^ B cells.** (a) IL-15 expression in peripheral blood cell types. The relative expression of IL-15 across immune cell types was analysed using the publicly available dataset GEO: GSE107019 as referenced (https://doi.org/10.1016/j.celrep.2019.01.041), and visualised in a bar chart. (b) IL-15 secretion of monocytes and CD8^+^ T cells in T1D patients and HC, as assessed by ELISA (n = 5). Student’s t-test was used for comparing two groups. (c) Representative flow cytometry plots and bar graphs of IL-15 expression in monocytes from T1D patients and HC (n = 7). Student’s t-test was used for comparing two groups. (d) Representative flow cytometry plots and bar graphs of IL-15 expression by macrophages in PLN from NOD mice and C57BL/6 mice (n = 6). Student’s t-test was used for comparing two groups. (e) Representative flow cytometry plots and line charts of CD226 expression on B cells isolated from T1D patients following 48-h culture with either medium control, 20 ng/ml IL-15R alpha & IL-15, 20 nM QNZ, or 20 ng/ml IL-15R alpha & IL-15 and 20 nM QNZ (n = 6). One-way repeated measures ANOVA followed by adjustments was used for multiple comparisons. Lines connect the same sample. (f and g) Representative flow cytometry plots and line charts of CD69 (f) and TNF-α (g) expression in CD226^+^ B cells from T1D patients after 48-h indicated stimulation (n = 6). One-way repeated measures ANOVA followed by adjustments was used for multiple comparisons. Lines connect the same sample. ∗P < 0.05. ∗∗P < 0.01. ∗∗∗P < 0.001. ∗∗∗∗P < 0.0001. Abbreviations: HC, healthy controls; T1D, type 1 diabetes; SSC-H, side scatter height; NOD, non-obese diabetic.

To explore the factors upstream of IL-15, further bioinformatics analysis revealed that the main source of IL-15 in the serum is peripheral monocytes in T1D patients (Fig. 6a). This result was supported by higher IL-15 secretion from monocytes in T1D patients than in HC, as detected by ELISA, with no differences observed in CD8^+^ T cells (Fig. 6b). We also found that higher IL-15 expression from monocytes in T1D patients than in HC, as shown by flow cytometry (Fig. 6c), with no differences observed in CD4^+^ T cells, CD8^+^ T cells, B cells, or NK cells (Supplementary Fig. S6a–d). Additionally, diabetic NOD mice expressed higher IL-15 in macrophages of PLN compared to C57BL/6 mice, while no difference was observed in CD8^+^ T cells, indicating that they may secrete more IL-15 (Fig. 6d, Supplementary Fig. S6e). Furthermore, we exposed purified CD226^+^ B cells to IL-15 with and without QNZ. We found that blocking NF-κB signalling led to decreased generation (Fig. 6e) and activation of CD226^+^ B cells (Fig. 6f), as well as reduction in the production of IL-15-induced cytokine by these cells (Fig. 6g).

### Blocking IL-15 signalling pathway prevents the onset and development of T1D in cyclophosphamide-induced NOD mice

**Fig. 7:**
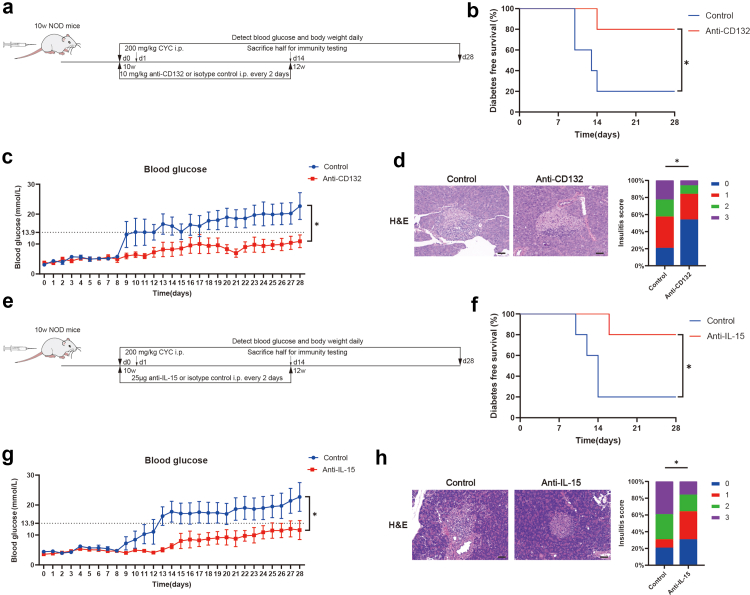
**Blocking IL-15 signalling pathway prevents cyclophosphamide-accelerated diabetes in NOD mice.** (a) Flowchart of in vivo anti-CD132 intervention in cyclophosphamide-accelerated NOD mice. (b) Survival curves of diabetes onset in the anti-CD132 group and isotype control group in cyclophosphamide-accelerated NOD mice (n = 5). Diabetes incidence was compared by the log-rank test for survival. (c) Blood glucose levels in the anti-CD132 group and isotype control group in cyclophosphamide-accelerated NOD mice (n = 5). Blood glucose levels were compared by two-way ANOVA. (d) Histopathological H&E staining images of pancreatic islets and insulitis scores from the anti-CD132 group and isotype control group in cyclophosphamide-accelerated NOD mice. Scale bar: 50 μm. Insulitis scores were compared by the chi-square test. (e) Flowchart of in vivo anti-IL-15 intervention in cyclophosphamide-accelerated NOD mice. (f) Survival curves of diabetes onset in the anti-IL-15 group and isotype control group in cyclophosphamide-accelerated NOD mice (n = 5). Diabetes incidence was compared by the log-rank test for survival. (g) Blood glucose levels in the anti-IL-15 group and isotype control group in cyclophosphamide-accelerated NOD mice (n = 5). Blood glucose levels were compared by two-way ANOVA. (h) Histopathological H&E staining images of pancreatic islets and insulitis scores from the anti-IL-15 group and isotype control group in cyclophosphamide-accelerated NOD mice. Scale bar: 50 μm. Insulitis scores were compared by the chi-square test. ∗P < 0.05. Abbreviations: NOD, non-obese diabetic; CYC, cyclophosphamide; i.p., intraperitoneally; H&E, haematoxylin and eosin.

To elucidate the prophylactic potential of anti-CD132 in T1D, we injected anti-CD132 or anti-IgG2b isotype control into nondiabetic NOD mice one day prior to cyclophosphamide injection (Fig. 7a). We found that anti-CD132 administration protected NOD mice from diabetes onset, hyperglycemia, and destructive insulitis compared with the isotype control group (Fig. 7b–d), with no difference in body weight (Supplementary Fig. S7a). Blockade of CD132 resulted in a reduction in the proportion of CD226^+^ B cells (Supplementary Fig. S7b). Moreover, the activation and cytokine production of B cells were reduced within the PLN after anti-CD132 administration (Supplementary Fig. S7c–e). Our findings revealed decreased expression of NF-κB signalling pathway target genes such as NIK, RELA, CXCR4 and CXCL10 within CD226^+^ B cells in the anti-CD132 group compared to the control group (Supplementary Fig. S7f). In addition, to enhance the specificity targeting the IL-15 signalling pathway, we conducted in vivo intervention using anti-IL-15 in cyclophosphamide-induced NOD mice to investigate its preventive effects (Fig. 7e). We found that anti-IL-15 administration prevented the onset and development of T1D in NOD mice (Fig. 7f–h), consistent with anti-CD132’s protective effects (Fig. 7a–d). Flow cytometry analysis revealed that blocking IL-15 also led to a reduction in the proportion of CD226^+^ B cells, as well as decreased B cell activation and cytokine production (Supplementary Fig. S7g–j). We also investigated the effect of anti-CD132 in T1D prevention using the STZ-induced diabetic mouse model (Supplementary Fig. S7k). The results showed that anti-CD132 administration protected the STZ-induced mice from diabetes onset and hyperglycemia (Supplementary Fig. S7l and m). In conclusion, our results underscore that blocking the IL-15 signalling pathway in T1D mouse models effectively reduces disease severity, highlighting CD132 as a prospective therapeutic target for T1D.

### Combination treatment with anti-CD3 and anti-CD132 synergistically reverts hyperglycemia in diabetic NOD mice

**Fig. 8:**
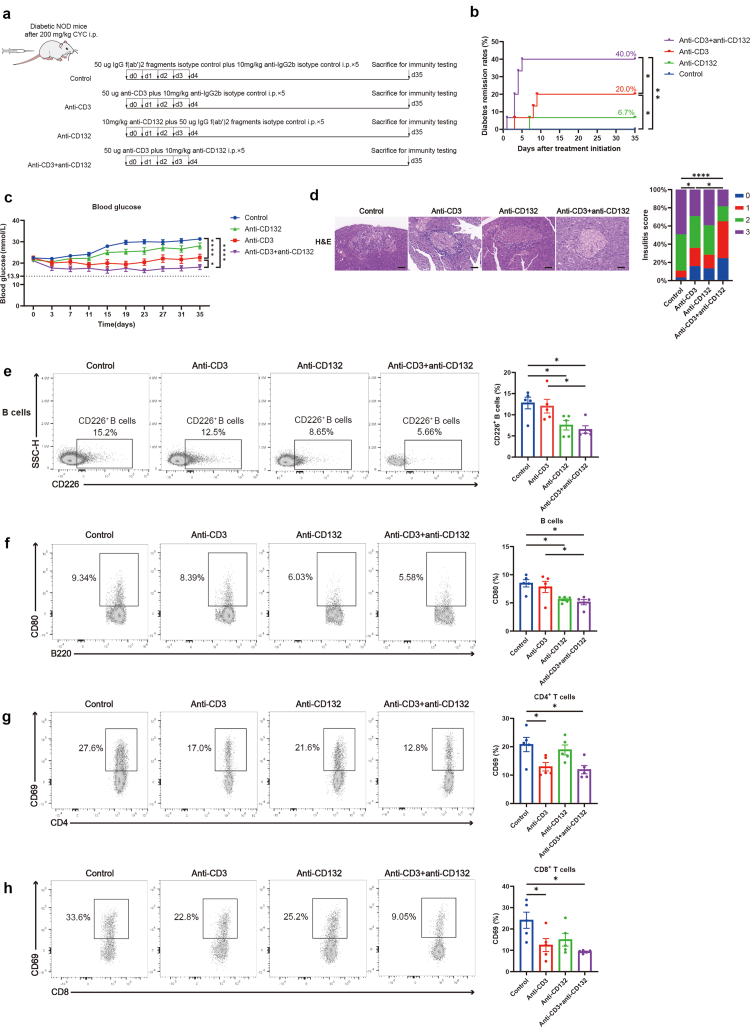
**Combination therapy with anti-CD3 and anti-CD132 synergistically reverts the type 1 diabetes in NOD mice.** (a) Experimental design for the four indicated groups. (b) Diabetes remission rates among the four indicated groups (n = 15). Diabetes remission rates were compared by cox proportional hazards regression test. (c) Blood glucose levels among the four indicated groups (n = 15). Blood glucose levels were compared by two-way ANOVA followed by Tukey’s multiple comparison test. (d) Histopathological H&E staining images of pancreatic islets and insulitis scores among the four indicated groups. Scale bar: 50 μm. Insulitis scores were compared by the chi-square test. (e) Representative flow cytometry plots and bar graphs of the proportions of CD226^+^ B cells in the PLN among the four indicated groups (n = 5). One-way ANOVA followed by adjustments was used for multiple comparisons. (f) Representative flow cytometry plots and bar graphs of the expression of CD80 in B cells in the PLN among the four indicated groups (n = 5). One-way ANOVA followed by adjustments was used for multiple comparisons. (g) Representative flow cytometry plots and bar graphs of the expression of CD69 in CD4^+^ T cells in the PLN among the four indicated groups (n = 5). One-way ANOVA followed by adjustments was used for multiple comparisons. (h) Representative flow cytometry plots and bar graphs of the expression of CD69 in CD8^+^ T cells in the PLN among the four indicated groups (n = 5). One-way ANOVA followed by adjustments was used for multiple comparisons. ∗P < 0.05. ∗∗P < 0.01. ∗∗∗∗P < 0.0001. Abbreviations: NOD, non-obese diabetic; CYC, cyclophosphamide; i.p., intraperitoneally; Anti-CD3, anti-CD3 monoclonal antibody; Anti-CD132, anti-CD132 monoclonal antibody; H&E, haematoxylin and eosin; SSC-H, side scatter height.

To evaluate the therapeutic potential of anti-CD132 in reversing diabetic NOD mice, we treated new-onset NOD mice with combination treatment of anti-CD3 plus anti-CD132, the respective monotherapies, or the control (Fig. 8a). We found that the diabetes remission rates were significantly higher for the mice treated with only anti-CD3 and combination therapy than for those in the control group. Combination therapy showed higher remission rates compared with that in only anti-CD3 group (Fig. 8b). The combination benefits were further evidenced by significantly reduced blood glucose and insulitis compared with those of anti-CD3 group, underscoring anti-CD132’s critical role in T1D treatment (Fig. 8c and d). Further flow cytometry revealed that combination-treated mice had significantly decreased CD226^+^ B cells percentage and activation of B cells in PLN compared with those in the control and anti-CD3 group (Fig. 8e and f). Combination treatment also decreased the percentage of pathogenic activated CD4^+^ T and CD8^+^ T cells compared with that in the control group (Fig. 8g and h). Collectively, anti-CD132 decreased pathogenic B cells, anti-CD3 decreased pathogenic T cells, and the combination treatment decreased both cell types. Anti-CD132 inhibited CD226^+^ B cells recruitment and activation of B cells, thereby improving the therapeutic efficacy of anti-CD3 immunotherapy in T1D.

## Discussion

While the roles of CD226^+^ CD8^+^ T cells in T1D have been previously reported, the importance and mechanism of CD226^+^ B cells remain poorly understood. Here, we identified the CD226^+^ B cell subset contributed to T1D pathogenesis, with an observed upregulation of CD226 associated with disease progression. Our findings demonstrated that CD226^+^ B cells exhibit enhanced activation and pro-inflammatory characteristics, along with a metabolic preference for glycolysis. Cellular experiments revealed that IL-15 secreted by monocytes or macrophages promotes the generation and functional capacity of CD226^+^ B cells. Notably, anti-CD132 or anti-IL-15 protected NOD mice from the cyclophosphamide-accelerated onset of hyperglycemia as a precaution. Furthermore, combination therapy with anti-CD3 and anti-CD132 in vivo synergistically reversed T1D. Together, this study highlights the pivotal role of the monocyte/macrophage-IL-15-CD226^+^ B cell axis in bridging innate with adaptive immunity in the development of T1D.

CD226, a member of the immunoglobulin-like superfamily, belongs to the activating receptor group that mediates cellular cytotoxicity signals.^23^ GWAS have demonstrated that polymorphisms in the CD226 gene are related to T1D susceptibility.^24^^,^^25^ It has been documented that the binding of CD226 on NK cells to its ligands CD112 and CD155 on target cells triggers downstream activation, which is vital for NK cell activation and cytotoxicity.^26^ Our research, along with studies from other laboratories, indicate that CD226^+^ CD8^+^ T cells constitute a highly cytotoxic subset involved in T1D progression.^19^^,^^27^ In this study, we identified elevated CD226 expression levels in circulating B cells and B cell subpopulations in T1D patients compared with HC, a phenomenon also observed in SLE patients.^18^ Clinical data analysis identified a positive relation between the proportion of CD226^+^ B cells and disease severity in T1D patients. LADA is a type of autoimmune diabetes (AID) that progresses more slowly compared with T1D patients, with gradual β-cell destruction.^28^ We did not observe elevated CD226^+^ B cell expression in T2D patients, despite their similarly hyperglycaemic environment, which suggests that LADA is more similar to T1D. We observed no associations between the proportion of CD226^+^ B cells and specific islet autoantibodies or their positive counts in T1D patients, possibly due to other factors influences like the age when symptoms first appeared and the duration of the illness.

The mechanisms underlying the generation of CD226^+^ B cells in T1D remain only partially explained. Utilising the bioinformatic analysis, our study suggested IL-15 as a key regulator of CD226 gene expression. IL-15 is known for promoting the growth, proliferation, maturation, and immunoglobulin production of B lymphocytes.^29^ Several studies have indicated that IL-15 contributes to the progression of autoimmune disorders like RA, inflammatory bowel disease, and coeliac disease.^30^^,^^31^ We and other study found that serum IL-15 levels were greater in T1D patients than HC,^32^ a trend similarly observed in SLE patients.^33^ In addition, we found that B cells in T1D patients highly expressed IL-15R γc (CD132) compared with HC, which may contribute to the upregulation of CD226^+^ B cells in T1D. We further demonstrated that elevated IL-15 promotes the upregulation of CD226 expression and stimulates B cell pro-inflammatory cytokine production. In line with previous studies, IL-15 is essential for maintaining the diabetes-promoting potential of antigen-presenting cells (APCs).^34^ We further elucidated that IL-15 signalling pathway blockade effectively reduces blood glucose levels and insulitis severity by attenuating inflammatory responses in B cells of NOD mice. Additionally, through bioinformatics analysis and experimental validation, we identified monocytes or macrophages as the source of IL-15. The innate immune system, including monocytes, is the first to be activated in T1D initial stages, orchestrating innate and adaptive immune activation in T1D.^35^^,^^36^ Our data indicated that monocytes or macrophages initiate IL-15 secretion, which subsequently activates adaptive immunity including CD226^+^ B cells, ultimately driving autoimmune destruction.

It is well established that the NF-κB signalling pathway plays vital roles in the regulation of immune and inflammatory responses. NF-κB increases the activation and autoantibody production of pathogenic B cells from SLE patients.^37^ NF-κB promotes the activation of immune cells and cytokine secretion, leading to disease exacerbation in RA.^38^^,^^39^ Furthermore, NF-κB activation in pancreatic β-cells results in β-cell damage in T1D,^40^ whereas NF-κB inhibition prevents STZ-induced T1D.^41^ Our study supports the indispensable role of NF-κB in mediating inflammatory cytokine production by CD226^+^ B cells. In addition, the use of NF-κB inhibitors effectively inhibited the activation of CD226^+^ B cells.

Although the anti-CD3 Teplizumab is the first Food and Drug Administration (FDA)-approved therapy for newly diagnosed T1D, it has not met expectations.^42^^,^^43^ This is evident in cases where some patients experience only transient stabilisation of islet function and others show no response for unidentified reasons, prompting further efforts to develop novel therapeutic strategies. Our results, along with previous studies,^44^^,^^45^ demonstrate that blocking CD132 shows efficacy in mitigating the progression of autoimmune diseases including T1D and SLE. Furthermore, we have shown that anti-CD132 holds promise both in the prevention and treatment of T1D, particularly having an additive effect with anti-CD3 treatment. Our study demonstrated that anti-CD3 in combination with anti-CD132 not only inhibits pathogenic T cells activation but also corrects the dysregulation of APCs including CD226^+^ B cells, which collectively contribute to the autoimmune process. These findings support the rationale of investigating the combination of anti-CD132 with teplizumab in new-onset T1D patients to potentially enhance clinical benefits.

The following limitations exist in our study. First, the sample capacity is comparatively limited. Before generalising the conclusions to a larger population, it is essential to duplicate the study using a more extensive sample size in multiple centres. Second, from a disease prediction perspective, determining the proportion and activity of CD226^+^ B cells to distinguish individuals who are positive for autoantibodies but have not yet progressed to overt T1D could be valuable. Given the importance of preventing T1D, future research could involve similar studies in high-risk susceptible populations. Third, our current findings demonstrate that IL-15 regulates the CD226^+^ B cells. Future studies will further elucidate the molecular pathway basis of IL-15-mediated regulation of CD226^+^ B cells. At last, anti-CD132 antibody inhibits the signalling of multiple cytokines such as IL-4, IL-7, and IL-9. Therefore, to address the specificity issue, we also conducted in vivo intervention experiments with anti-IL-15 antibody, which showed effects comparable to those of anti-CD132.

**Fig. 9:**
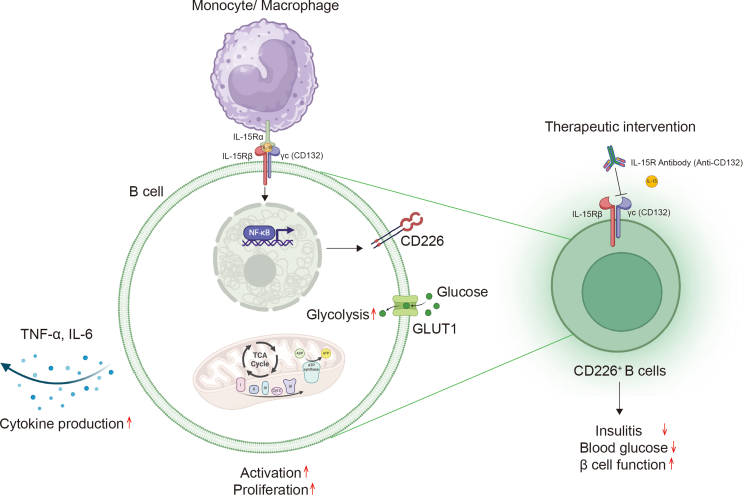
**Working model of the monocyte/macrophage-IL-15-CD226^+^ B cell axis in the immunopathogenesis of T1D.** There is increased secretion of IL-15 by monocytes or macrophages in T1D. IL-15 secreted by monocytes or macrophages binds to IL-15Rβ/γc on B cells. This further enhances the expression of CD226 on B cell surfaces and increases their pro-inflammatory cytokine production, activation, and proliferative capacity. The NF-κB signalling pathway promotes the generation and pro-inflammatory responses of CD226^+^ B cells. Targeting the IL-15-IL-15R signalling pathway reverses inflammatory phenotypes, mitigating disease severity, thereby representing a promising therapeutic strategy for preventing T1D. Abbreviations: T1D, type 1 diabetes; GLUT1, glucose transporter type 1.

In conclusion, the findings of this study provide new insights into B cell-related immunotherapy for T1D patients. Targeting the monocyte/macrophage-IL-15-CD226^+^ B cell axis may provide an effective strategy for treating T1D (Fig. 9).

## Contributors

Z.Z., B.Z., J.H. (Jiaqi Huang)., and J.L. conceived and designed the structure of the original research. J.L. and X.L. collected the clinical samples and performed the experiments. J.L. analysed the data, created the figures, and drafted the manuscript. B.Z., J.L., M.Z., W.L., J.H. (Juan Huang) and Y.X. commented and revised the manuscript. All the authors contributed to the article and approved the submitted version.

## Data sharing statement

All relevant data are included in the main text, figures, tables, and Supplementary materials. Data from this study is available from the corresponding author upon reasonable request.

## Declaration of interests

The authors declared that they have no conflict of interest.
