# Supplementary material for: Monocyte/macrophage-derived interleukin-15 mediates the pro-inflammatory phenotype of CD226+ B cells in type 1 diabetes
Source: eBioMedicine. 2025 Sep 15;120:105946. doi: 10.1016/j.ebiom.2025.105946 (PMC12466144; doi:10.1016/j.ebiom.2025.105946)
Supplement: Supplementary Figs. S1–S7 and Tables S1 and S2 [file mmc1.doc]

**Supplementary Figure**


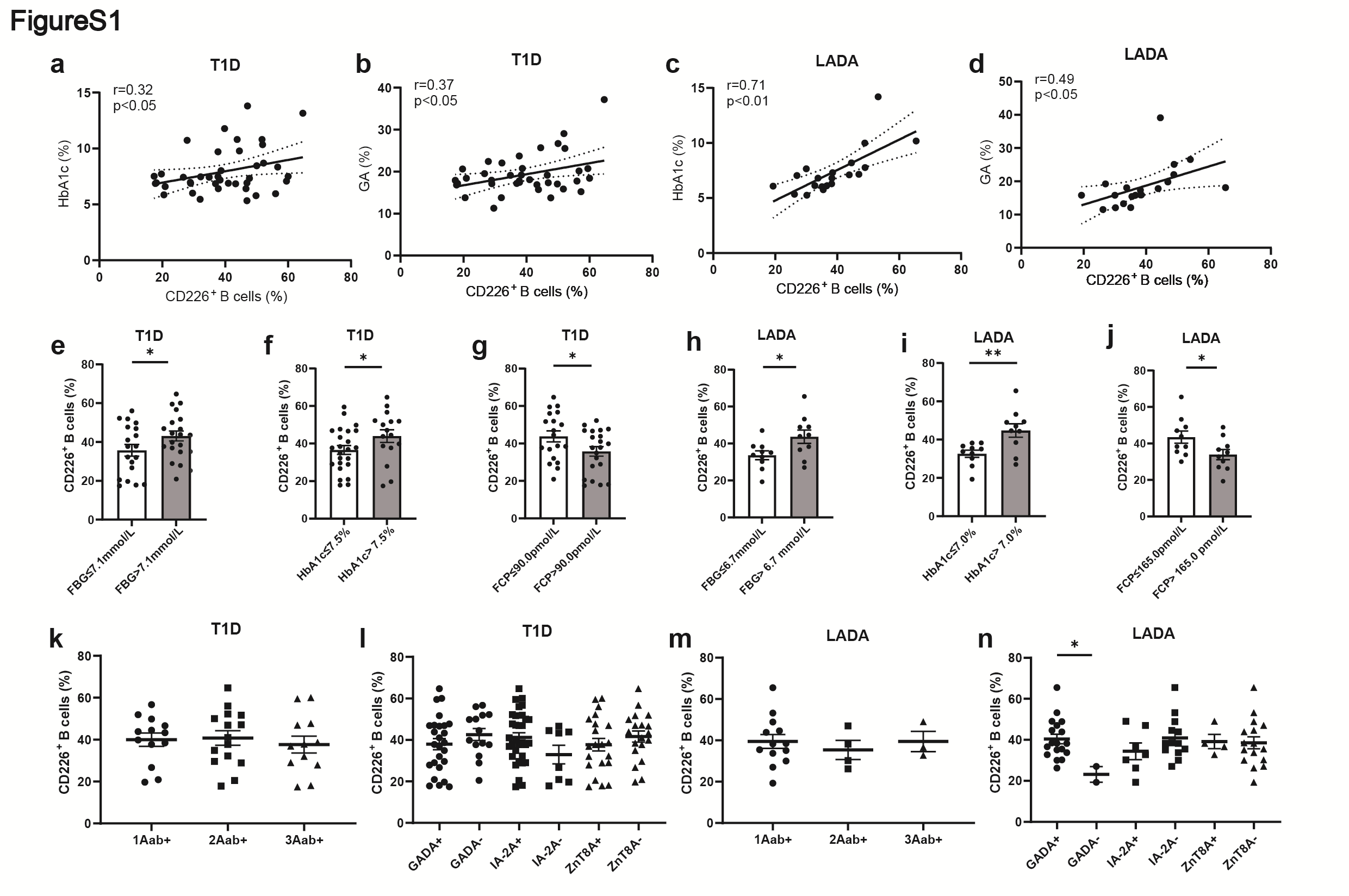


**Supplementary Figure S1: Analysis of the proportion of CD226^+^ B cells in relation to the clinical characteristics of T1D patients.**

(a-d) Relationships between CD226^+^ B cells and clinical parameters from T1D (n = 40) and LADA (n = 20). Correlations between the percentage of CD226^+^ B cells and HbA1c (a), GA (b) in T1D patients. Correlations between the percentage of CD226^+^ B cells and HbA1c (c), GA (d) in LADA patients. Pearson or Spearman's rank correlation was used for correlation analyses. Linear regression is shown with 95% CIs (dotted area). (e-j) The percentage of CD226^+^ B cells is related to clinical features of T1D (n = 40) and LADA (n = 20). T1D patients with FBG ＞ 7.1 mmol/L (e), HbA1c ＞ 7.5% (f), and FCP ≤ 90.0 pmol/L (g) had higher percentage of CD226^+^ B cells. LADA patients with FBG ＞ 6.7 mmol/L (h), HbA1c ＞ 7.0% (i), and FCP ≤ 165.0 pmol/L (j) had higher percentage of CD226^+^ B cells. Student’s t-test was used for comparing two groups. Each point represents an individual. Horizontal bars represent the mean ± SEM. (k-n) Relationships between CD226^+^ B cells and autoantibodies in T1D (n = 40) and LADA (n = 20). The relationships between the count of positive autoantibodies and the percentage of CD226^+^ B cells in both T1D (k) and LADA (m) were examined. The relationships between the positive or negative results for GADA, IA-2A, and ZnT8A and the percentage of CD226^+^ B cells in both T1D (l) and LADA (n) were examined. One-way ANOVA followed by adjustments was used for multiple comparisons. Student’s t-test was used for comparing two groups.*P < 0.05. **P < 0.01. Abbreviations: T1D, type 1 diabetes; LADA, latent autoimmune diabetes in adults; HbA1c, glycated hemoglobin; GA, glycated albumin; FBG, fasting blood glucose; FCP, fasting C-peptide; Aab, autoantibody; GADA, glutamic acid decarboxylase antibody; IA-2A, protein tyrosine phosphatase antibody; ZnT8A, zinc transporter 8 antibody.


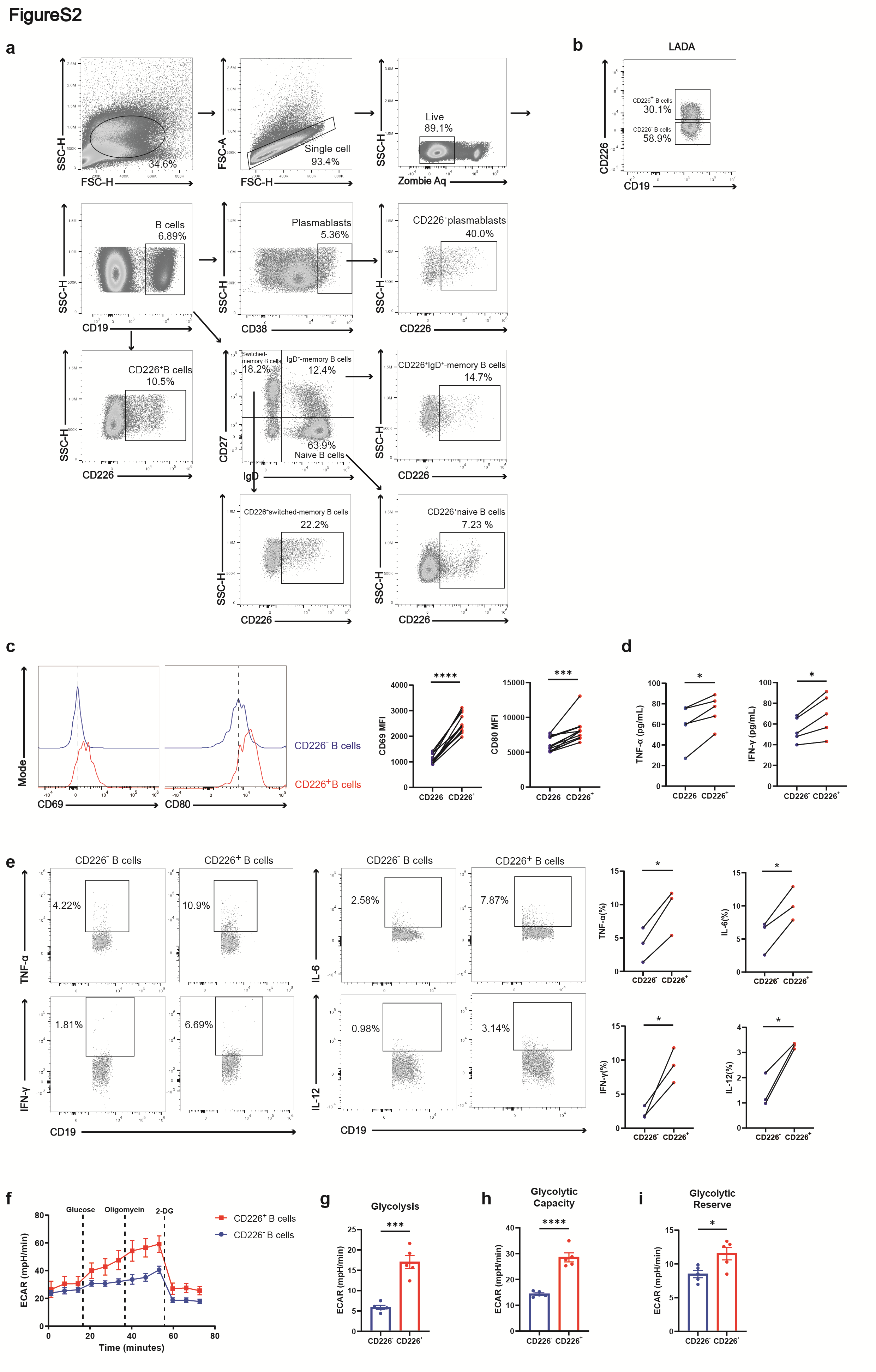


**Supplementary Figure S2: Functional and metabolic analysis of CD226^+^ B cells and CD226^-^ B cells in human.**

(a) Flow cytometry analysis and gating strategy applied to human peripheral B cell subsets for CD226 expression. After gating on total lymphocytes, CD19^+^ B cells were selected on the basis of single live cells. The following subpopulations were subsequently derived from the CD19^+^ B cells gate: naive B cells, IgD^+^-memory (IgD^+^-m) B cells, switched-memory (SM) B cells, and plasmablasts. CD226 expression was detected in all of these subpopulations. The B cell subsets were defined as follows: B cells (CD19^+^), naive B cells (CD19^+^IgD^+^CD27^−^), IgD^+^-memory B cells (CD19^+^IgD^+^CD27^+^), switched-memory (SM) B cells (CD19^+^IgD^−^CD27^+^), and plasmablasts (CD19^+^CD38^++^). (b) Peripheral blood B cells were categorized into CD226^+^ and CD226^-^ B cells on the basis of CD226 expression in LADA. (c) Representative flow cytometry plots and scatter plots for paired t-tests of CD69 and CD80 expression in CD226^+^ and CD226^-^ B cells from HC (n = 12). (d) TNF-α and IFN-γ secretion of CD226^+^ and CD226^-^ B cells from T1D patients, as assessed by ELISA (n = 5). A paired t-test was used for comparing two groups. (e) Representative flow cytometry plots and scatter plots for paired t-tests of TNF-α, IFN-γ, IL-6, and IL-12 expression in CD226^+^ and CD226^-^ B cells from LADA patients (n = 3). (f) The ECAR curve of CD226^+^ B cells and CD226^-^ B cells isolated from T1D patients (n = 5). (g-i) The bar graphs of ECAR of CD226^+^ B cells and CD226^-^ B cells isolated from T1D patients (n = 5). Glycolysis was calculated by ECAR after addition of glucose subtracting ECAR before glucose addition (g). Glycolytic capacity was determined by ECAR after addition of oligomycin subtracting ECAR before glucose addition (h). Glycolytic reserve was glycolytic capacity subtracting glycolysis (i). Student’s t-test was used for comparing two groups. *P < 0.05. ***P < 0.001. ****P < 0.0001. Abbreviations: IgD^+^-m B cells, IgD^+^-memory B cells; SM B cells, switched memory B cells; SSC-H, side scatter height; FSC-H, forward scatter height; FSC-A, forward scatter area; LADA, latent autoimmune diabetes in adults; HC, healthy controls; T1D, type 1 diabetes; ECAR, extracellular acidification rate.


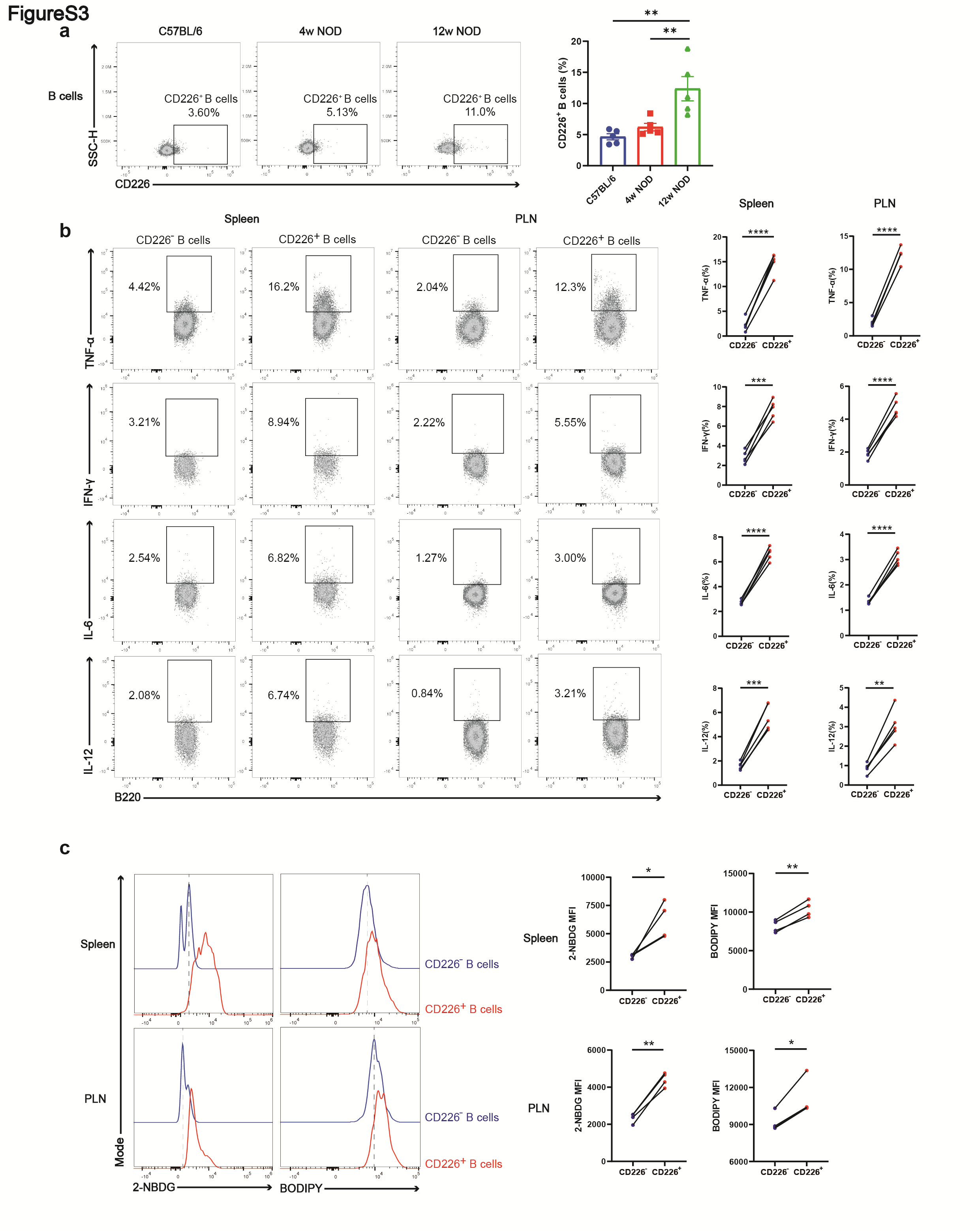


**Supplementary Figure S3: Functional and metabolic analysis of CD226^+^ B cells and CD226^-^ B cells in NOD mice.**

(a) The expression levels of CD226 on B cells in peripheral blood at 4 and 12 weeks of age in NOD mice and 12 weeks of age in C57BL/6 mice (n = 5). One-way ANOVA followed by adjustments was used for multiple comparisons. (b-c) Representative flow cytometry plots and scatter plots for paired t-tests of TNF-α, IFN-γ, IL-6, IL-12 (n = 5) (b), 2-NBDG, and BODIPY (n = 4) (c) expression in CD226^+^ and CD226^-^ B cells from the spleen and PLN of NOD mice. *P < 0.05. **P < 0.01. ***P < 0.001. ****P < 0.0001. Abbreviations: NOD, non-obese diabetic; PLN, pancreatic lymph node; 2-NBDG, 2-deoxy-2-[(7-nitro-2,1,3-benzoxadiazol-4-yl)amino]-D-glucose; BODIPY, boron-dipyrromethene.


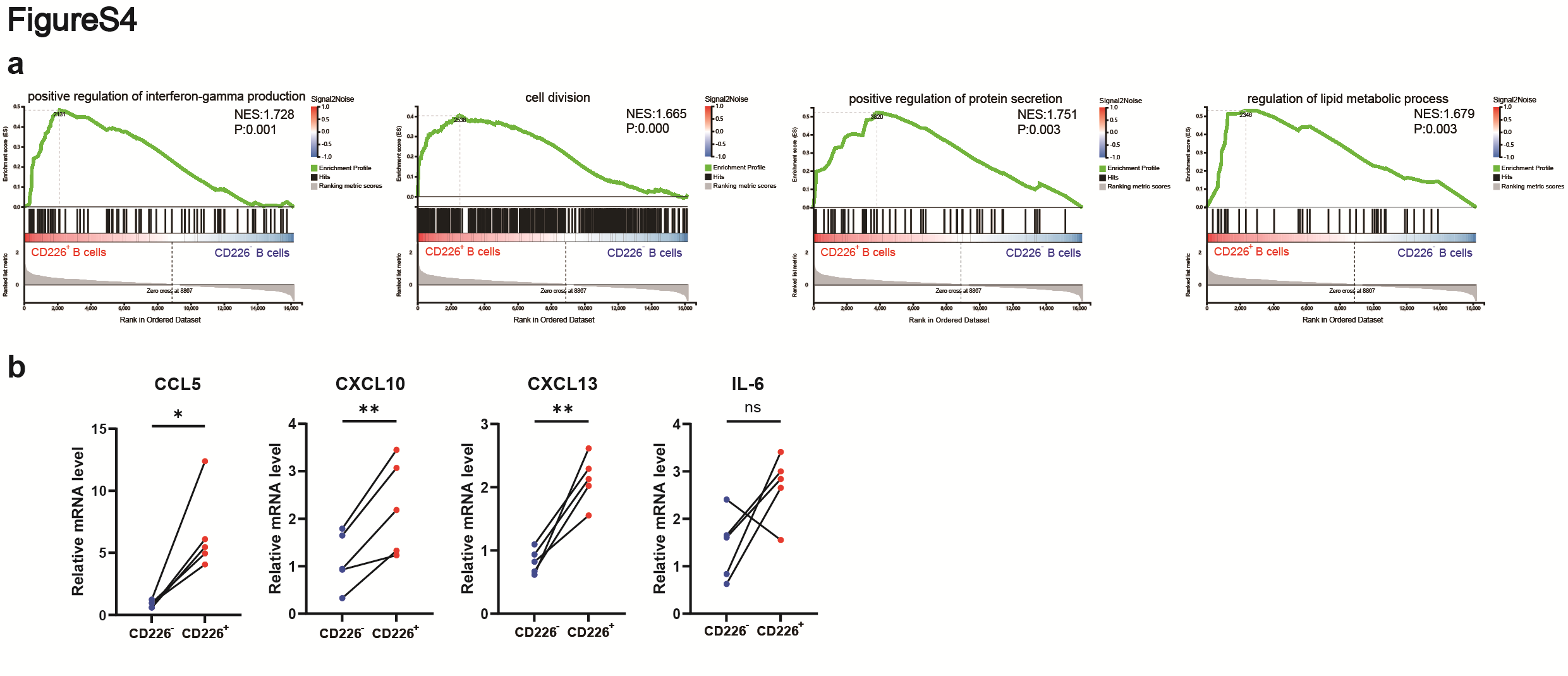


**Supplementary Figure S4: Enhanced inflammatory responses of CD226^+^ B cells via** **NF-κB signaling.**

(a) GSEA for positive regulation of interferon-gamma production, cell division, positive regulation of protein secretion, and regulation of lipid metabolic process associated genes in CD226^+^ B cells versus CD226^-^ B cells. (b) Quantitative real-time PCR (qPCR) analysis of NF-κB target genes expression in CD226^+^ B cells and CD226^-^ B cells from NOD mice (n = 5). A paired t-test was used for comparing two groups. *P < 0.05. **P < 0.01. Abbreviations: GSEA, gene set enrichment analysis; NOD, non-obese diabetic; ns, not significant.


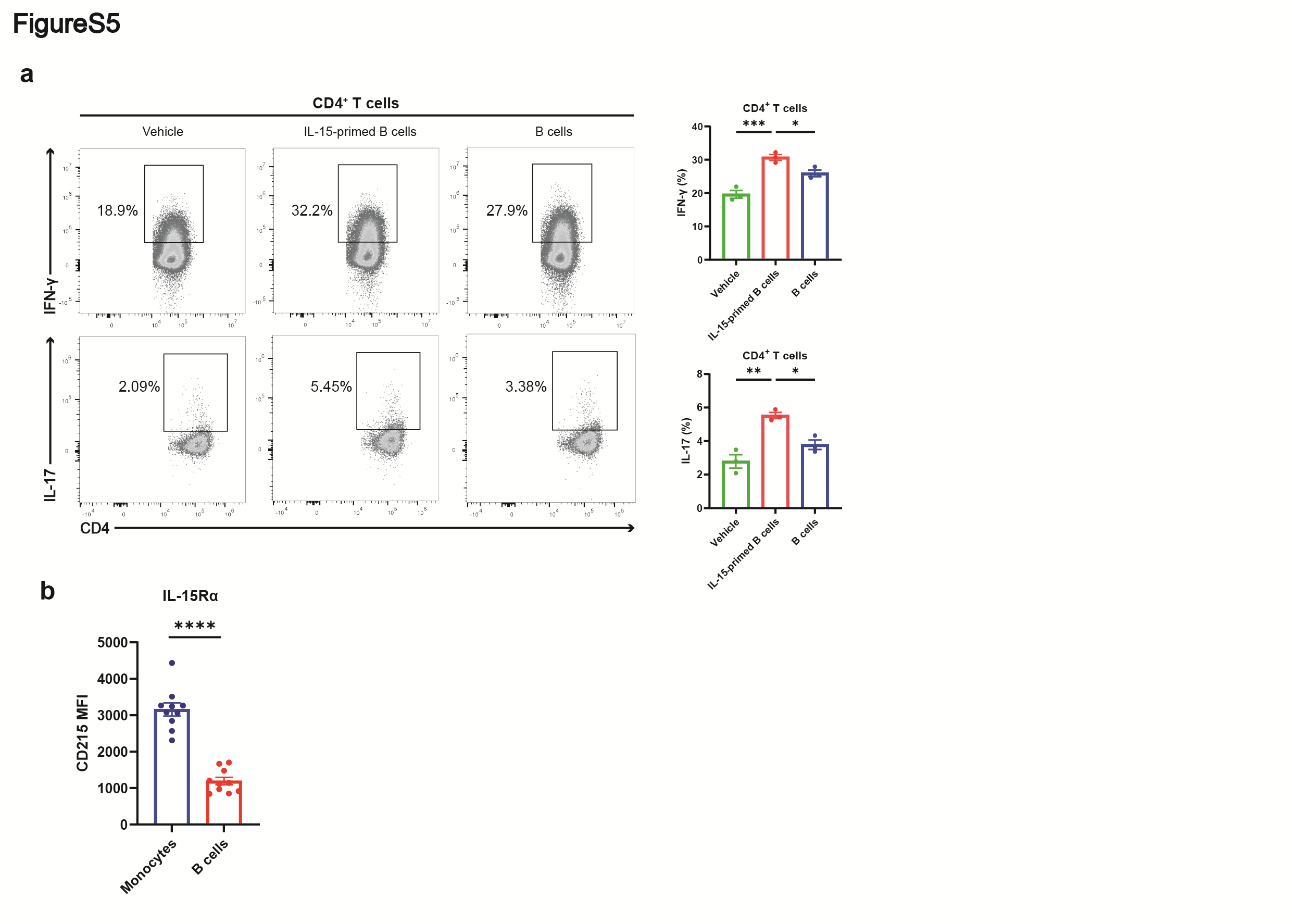


**Supplementary Figure S5: IL-15-primed B cells promote the activation of autoreactive T cells.**

(a) Representative flow cytometry plots and bar graphs of CD4^+^ T cell responses to IL-15-primed B cells or B cells in vitro (n = 3). One-way ANOVA followed by adjustments was used for multiple comparisons. (b) The expression of IL-15Rα (CD215) in monocytes and B cells from T1D patients (n = 10). Student’s t-test was used for comparing two groups. *P < 0.05. **P < 0.01. ***P < 0.001. ****P < 0.0001. Abbreviations: T1D, type 1 diabetes.


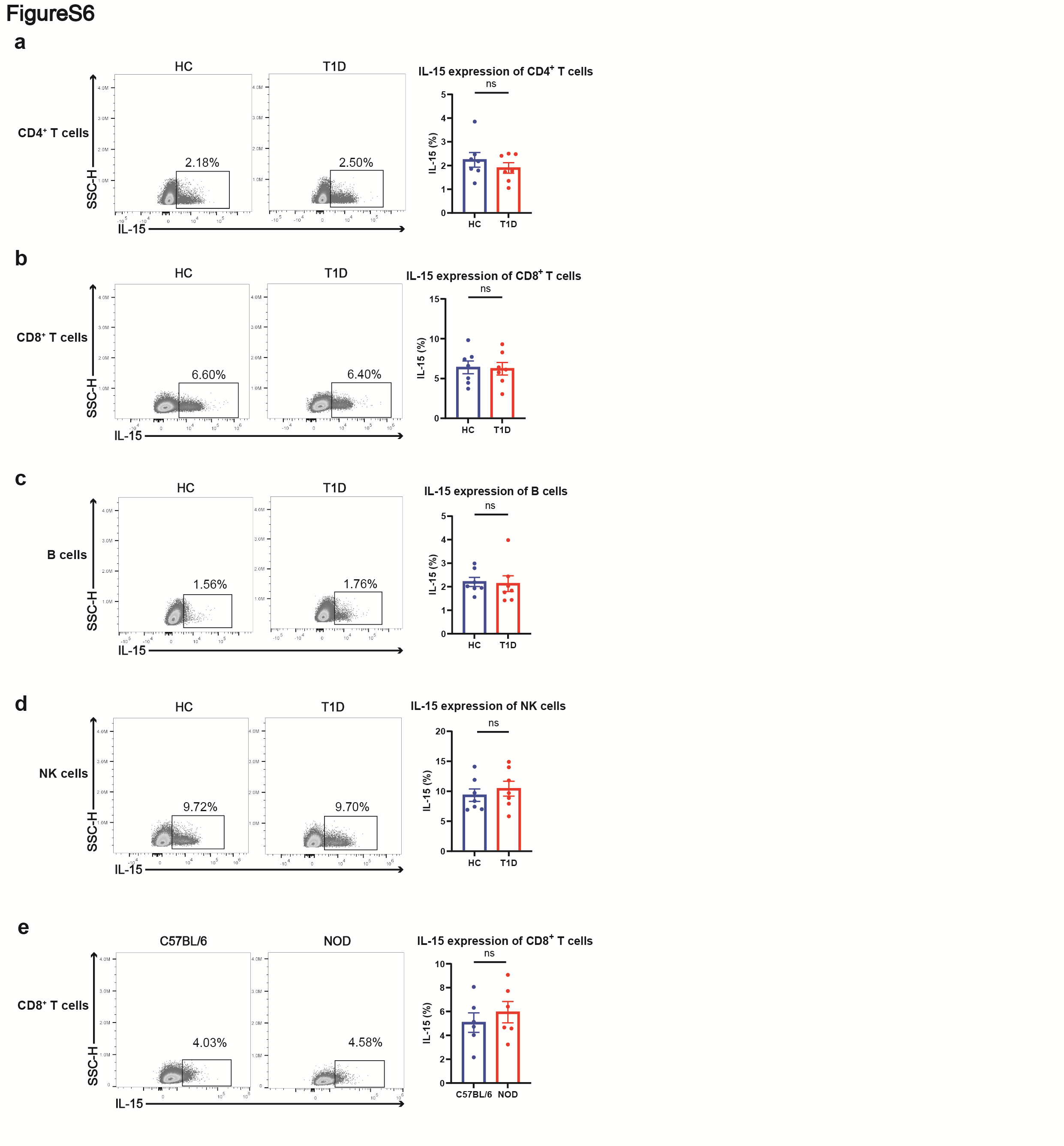


**Supplementary Figure S6: The expression of IL-15 in peripheral immune cells.**

(a-d) Representative flow cytometry plots and bar graphs of IL-15 expression in CD4^+^ T cells (a), CD8^+^ T cells (b), B cells (c), and NK cells (d) from T1D patients and HC (n = 7). Student’s t-test was used for comparing two groups. (e) Representative flow cytometry plots and bar graphs of IL-15 expression by CD8^+^ T cells in PLN from NOD mice and C57BL/6 mice (n = 6). Student’s t-test was used for comparing two groups. Abbreviations: HC, healthy control; T1D, type 1 diabetes; NK, natural killer; NOD, non-obese diabetic; SSC-H, side scatter height; ns, not significant.


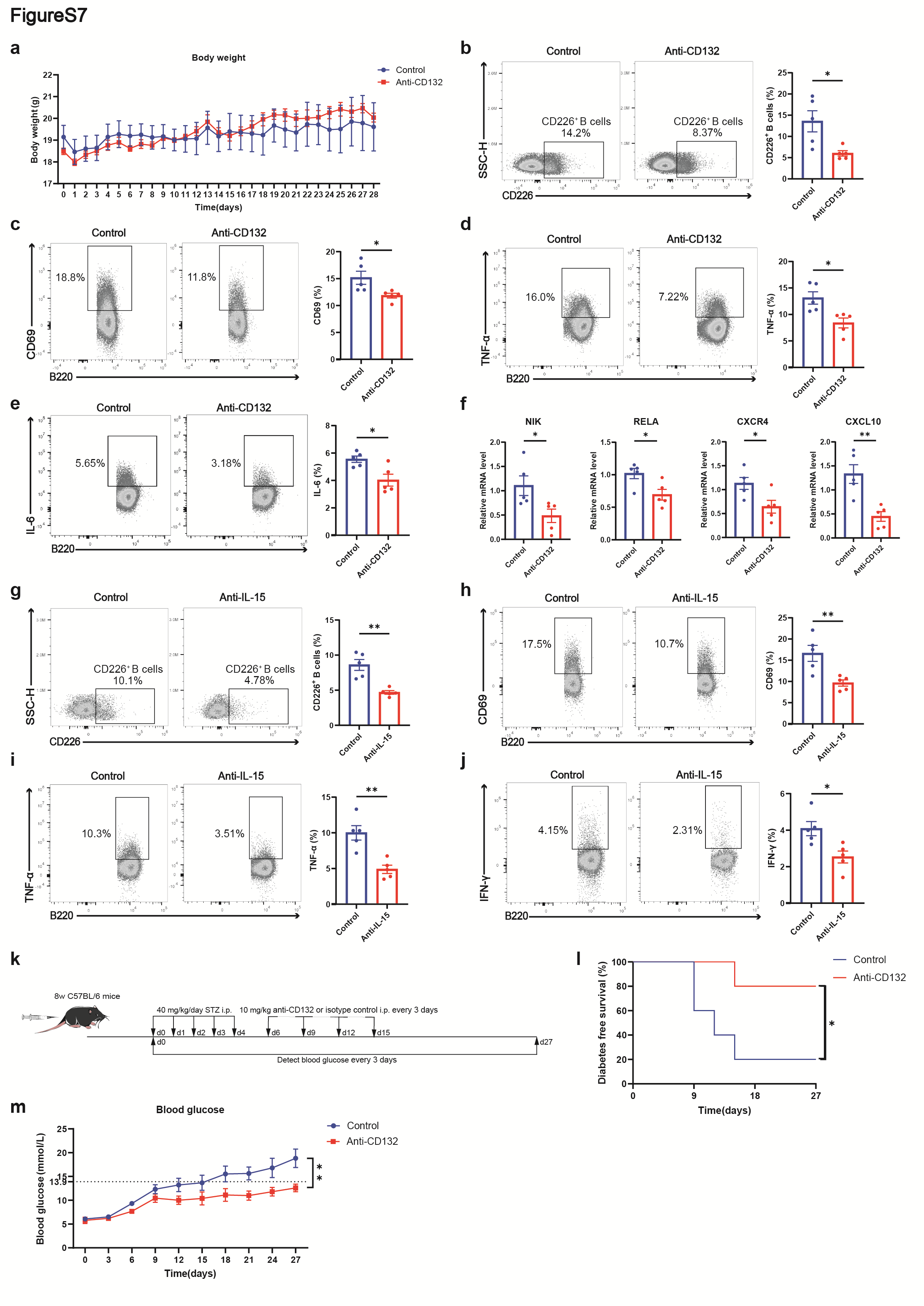


**Supplementary Figure S7:** **Blocking IL-15 signaling pathway prevents the onset and development of T1D.**

(a) Body weight levels in the anti-CD132 group and isotype control group in cyclophosphamide-accelerated NOD mice (n = 5). Body weight levels were compared by two-way ANOVA followed by Tukey’s multiple comparison test. (b) Representative flow cytometry plots and bar graphs of the proportions of CD226^+^ B cells in the PLN of anti-CD132 group and isotype control group in cyclophosphamide-accelerated NOD mice (n = 5). Student’s t-test was used for comparing two groups. (c-e) Representative flow cytometry plots and bar graphs of the expression of CD69 (c), TNF-α (d), and IL-6 (e) in B cells in the PLN between anti-CD132 group and isotype control group in cyclophosphamide-accelerated NOD mice (n = 5). Student’s t-test was used for comparing two groups. (f) Quantitative real-time PCR (qPCR) analysis for NF-κB target genes expression in CD226^+^ B cells from spleen of anti-CD132 group and isotype control group in cyclophosphamide-accelerated NOD mice (n = 5). Student’s t-test was used for comparing two groups. (g) Representative flow cytometry plots and bar graphs of the proportions of CD226^+^ B cells in the PLN of anti-IL-15 group and isotype control group in cyclophosphamide-accelerated NOD mice (n = 5). Student’s t-test was used for comparing two groups. (h-j) Representative flow cytometry plots and bar graphs of the expression of CD69 (h), TNF-α (i), and IFN-γ (j) in B cells in the PLN between anti-IL-15 group and isotype control group in cyclophosphamide-accelerated NOD mice (n = 5). Student’s t-test was used for comparing two groups. (k) Flowchart of in vivo anti-CD132 intervention in STZ-induced diabetic mouse model. (l) Survival curves of diabetes onset in the anti-CD132 group and isotype control group in STZ-induced diabetic mouse model (n = 5). Diabetes incidence was compared by the log-rank test for survival. (m) Blood glucose levels in the anti-CD132 group and isotype control group in STZ-induced diabetic mouse model (n = 5). Blood glucose levels were compared by two-way ANOVA. *P < 0.05. **P < 0.01. Abbreviations: NOD, non-obese diabetic; STZ, streptozotocin; i.p., intraperitoneally; SSC-H, side scatter height.

**Supplementary Table S1: List of all fluorescent-labeled anti-human and anti-mouse monoclonal antibodies.**

| Antibody | Source | | Identifier  (catalog number) | RRID | Dilution |
| --- | --- | --- | --- | --- | --- |
| anti-human-CD19-BB700 | BD | | Cat#566396 | AB_2744310 | 1:100 |
| anti-human-CD19-PerCP/Cyanine (Cy) 5.5 | Biolegend | | Cat#302230 | AB_2073119 | 1:100 |
| anti-human-CD19-Brilliant Violet (BV)605 | BD | | Cat#562653 | AB_2722592 | 1:100 |
| anti-human-IgD-Brilliant Violet (BV) 421 | BioLegend | | Cat#348226 | AB_2561619 | 1:100 |
| anti-human-CD27-APC | BioLegend | | Cat#356410 | AB_2561957 | 1:100 |
| anti-human-CD38-PE/Cyanine (Cy) 7 | BioLegend | | Cat#303516 | AB_2072782 | 1:100 |
| anti-human-CD226-PE | BioLegend | | Cat#338306 | AB_2275498 | 1:100 |
| anti-human-CD226-Brilliant Violet (BV) 711 | BioLegend | | Cat#338334 | AB_2728304 | 1:100 |
| anti-human-CD69-PE/Cyanine (Cy) 5 | BD | | Cat#555532 | AB_395917 | 1:100 |
| anti-human-CD80-PE/Dazzle 594 | BioLegend | | Cat#305229 | AB_2566488 | 1:100 |
| anti-human-CD86-APC | BD | | Cat#560956 | AB_10563076 | 1:100 |
| anti-human-HLA-DR-Alexa Fluor 700 | BD | | Cat#560743 | AB_1727526 | 1:100 |
| anti-human-TNF-α-PE/Dazzle 594 | BioLegend | | Cat#502946 | AB_2564173 | 1:100 |
| anti-human-TNF-α-APC | BD | | Cat#554514 | AB_398566 | 1:100 |
| anti-human-IFN-γ-Brilliant Violet (BV) 711 | BioLegend | | Cat#502540 | AB_2563506 | 1:100 |
| anti-human-IFN-γ-PerCP/Cyanine (Cy) 5.5 | BD | | Cat#560704 | AB_1727532 | 1:100 |
| anti-human-IL-6-PE/Dazzle 594 | BioLegend | | Cat#501122 | AB_2810622 | 1:100 |
| anti-human-IL-12-PerCP-eFluor 710 | eBioscience | | Cat#46-7235-42 | AB_1907423 | 1:100 |
| anti-human-CD215 (IL-15Rα)-FITC | eBioscience | | Cat#11-7159-42 | AB_2572516 | 1:100 |
| anti-human-CD122 (IL-2Rβ)-APC | BioLegend | | Cat#339007 | AB_2248891 | 1:100 |
| anti-human-CD132 (commonγchain)-PE | BioLegend | | Cat#338605 | AB_1279079 | 1:100 |
| anti-human-IL-15-APC | | Thermo Fisher Scientific | Cat#MA5-23627 | AB_2608838 | 1:10 |
| anti-human-CD14-Alexa Fluor 700 | BD | | Cat#557923 | AB_396944 | 1:100 |
| anti-human-CD3-APC/Cyanine (Cy) 7 | BioLegend | | Cat#344818 | AB_10645474 | 1:100 |
| anti-human-CD4-BB515 | BD | | Cat#564419 | AB_2744419 | 1:100 |
| anti-human-CD8-Brilliant Violet (BV) 650 | BD | | Cat#563821 | AB_2744462 | 1:100 |
| anti-human-CD56-PE/CF594 | BD | | Cat#562289 | AB_11152080 | 1:100 |
| anti-mouse-CD45-Brilliant Violet (BV) 750 | BioLegend | | Cat#103157 | AB_2734155 | 1:100 |
| anti-mouse-CD11b-RB744 | BD | | Cat#570512 | AB_3665015 | 1:100 |
| anti-mouse-B220-PerCP/Cyanine (Cy) 5.5 | BioLegend | | Cat#103235 | AB_893356 | 1:100 |
| anti-mouse-CD3-APC/Cyanine (Cy) 7 | BD | | Cat#557596 | AB_396759 | 1:100 |
| anti-mouse-CD4-Brilliant Violet (BV) 605 | BD | | Cat#563151 | AB_2687549 | 1:100 |
| anti-mouse-CD8-BB515 | BD | | Cat#564422 | AB_2738801 | 1:100 |
| anti-mouse-CD226-Brilliant Violet (BV) 605 | BioLegend | | Cat#133613 | AB_2715976 | 1:100 |
| anti-mouse-CD226-Brilliant Violet (BV) 650 | BioLegend | | Cat#133621 | AB_2716083 | 1:100 |
| anti-mouse-CD40-PE | BioLegend | | Cat#124610 | AB_1134075 | 1:100 |
| anti-mouse-CD69-PE/Dazzle 594 | BD | | Cat#562455 | AB_11154217 | 1:100 |
| anti-mouse-CD80-PE/Cyanine (Cy) 7 | BioLegend | | Cat#104734 | AB_2563113 | 1:100 |
| anti-mouse-CD86-APC | BioLegend | | Cat#105012 | AB_493342 | 1:100 |
| anti-mouse-I-A/I-E-Alexa Fluor 700 | BioLegend | | Cat#107622 | AB_493727 | 1:100 |
| anti-mouse-TNF-α-PE | BioLegend | | Cat#506306 | AB_315427 | 1:100 |
| anti-mouse-IFN-γ-Brilliant Violet (BV) 711 | BioLegend | | Cat#505836 | AB_2650928 | 1:100 |
| anti-mouse-IL-6-APC | BioLegend | | Cat#504508 | AB_10694868 | 1:100 |
| anti-mouse-IL-12-PE/Cyanine (Cy) 7 | BioLegend | | Cat#505209 | AB_2565644 | 1:100 |
| anti-mouse-Ki-67-PerCP-eFluor 710 | eBioscience | | Cat#46-5698-82 | AB_11040981 | 1:100 |
| anti-mouse-IL-17A-Brilliant Violet (BV) 785 | BioLegend | | Cat#506928 | AB_2629787 | 1:100 |
| Alexa Fluor® 488 Anti-IL-15 antibody | Abcam | | Cat#ab322228 | Not Available | 1:50 |
| Zombie Aqua™ Fixable Viability Kit | BioLegend | | Cat#423101 | Not Available | 1:100 |

**Supplementary Table S2: Human and mouse primer sequences for qRT-PCR.**

| Human [primer](https://www.baidu.com/link?url=eOGl6j2fFE-zkHIYE4vMVPgKInqDwPT-tQ-JgFO-eLe7VhGwgI_5HLtLgrqKzDx9TP70lfw9g3Q4hRQsCILbKmGVJGtfln1Ucfj8RJhf4S3&wd=&eqid=85a414ac0003478a0000000664d3855f) | Primer sequence (5' to 3') |
| --- | --- |
| ACTIN-F | CCTGGCACCCAGCACAAT |
| ACTIN-R | GGGCCGGACTCGTCATAC |
| NIK-F | GGCTGCTGTGAGAGTGTCAAGTG |
| NIK-R | TAACCTACGGGCGGGCATACC |
| TNF-F | AAGGACACCATGAGCACTGAAAGC |
| TNF-R | AGGAAGGAGAAGAGGCTGAGGAAC |
| CCL5-F | CCCGAAAGAACCGCCAAGTG |
| CCL5-R | GACTCTCCATCCTAGCTCATCTCC |
| CXCL10-F | AAAGAAGGGTGAGAAGAGATGTCTG |
| CXCL10-R | AGACCTTTCCTTGCTAACTGCTTTC |
| IL-6-F | GTGTTGCCTGCTGCCTTCC |
| IL-6-R | TCTGAAGAGGTGAGTGGCTGTC |
| RELA-F | ACAGAAGCAGGCTGGAGGTAAGG |
| RELA-R | GGACAATGCCAGTGCCATACAGG |

| Mouse [primer](https://www.baidu.com/link?url=eOGl6j2fFE-zkHIYE4vMVPgKInqDwPT-tQ-JgFO-eLe7VhGwgI_5HLtLgrqKzDx9TP70lfw9g3Q4hRQsCILbKmGVJGtfln1Ucfj8RJhf4S3&wd=&eqid=85a414ac0003478a0000000664d3855f) | Primer sequence (5' to 3') |
| --- | --- |
| GAPDH-F | GCAAATTCAACGGCACAGTCAAG |
| GAPDH-R | TCGCTCCTGGAAGATGGTGATG |
| CCL5-F | CCACTCCCTGCTGCTTTGC |
| CCL5-R | ACTTGCTGCTGGTGTAGAAATACTC |
| CXCL10-F | GCCTCATCCTGCTGGGTCTG |
| CXCL10-R | TCATTCTCACTGGCCCGTCATC |
| CXCL13-F | TCCTCGTGCCAAATGGTTACAAAG |
| CXCL13-R | TCAGGCAGCTCTTCTCTTACTCAC |
| IL-6-F | GTTGCCTTCTTGGGACTGATGC |
| IL-6-R | GGTATCCTCTGTGAAGTCTCCTCTC |
| NIK-F | AGCCTCAGCCTCCTCTACCG |
| NIK-R | GCCAGACTCCTCCTTGCTCAG |
| RELA-F | GTATTGCTGTGCCTACCCGAAAC |
| RELA-R | GCTGAGGGATGCTGGGAAGG |
| CXCR4-F | ACGCCACCAACAGTCAGAGG |
| CXCR4-R | TGAAGTCAGGTATAGTCAGGAGGAG |
